# Epoxy metabolites of linoleic acid promote the development of breast cancer via orchestrating PLEC/NFκB1/CXCL9-mediated tumor growth and metastasis

**DOI:** 10.1038/s41419-024-07300-6

**Published:** 2024-12-18

**Authors:** Kai-Di Ni, Xian Fu, Ying Luo, Xin He, Hou-Hua Yin, Dong-Ping Mo, Jing-Xian Wu, Ming-Jun Wu, Xiao Zheng, Ya-Nan Liu, Qing Jiang, Ling-Tong Zhang, Ai-Zhi Lin, Ling Huang, Qing-Jin Pan, Xue-Dong Yin, Huan-Yu Zhang, Yi-Wen Meng, Xue Zhou, Jianbo Pan, Zufeng Guo, Jun-Yan Liu

**Affiliations:** 1 CNTTI of College of Pharmacy and Department of Anesthesia of the Second Affiliated Hospital, Chongqing Medical University, Chongqing, China; 2 Basic Medicine Research and Innovation Center for Novel Target and Therapeutic Intervention, Ministry of Education, Chongqing, China; 3 Department of Clinical Laboratory, Shanghai Changning Maternity and Infant Health Hospital, East China Normal University, Shanghai, China; 4 Department of Clinical Laboratory, Jiangsu Cancer Hospital, the Affiliated Cancer Hospital of Nanjing Medical University, Jiangsu Institute of Cancer Research, Nanjing, China; 5 Department of Pathology, College of Basic Medicine of Chongqing Medical University, Chongqing, China; 6 Molecular Medicine Diagnostic and Testing Center, Chongqing Medical University, Chongqing, China; 7 Department of Pathology, The First Affiliated Hospital of Chongqing Medical University, Chongqing, China; 8 Center for Science & technology Innovation, Chongqing Medical University, Chongqing, China; 9 Center for Novel Target and Therapeutic Intervention (CNTTI), College of Pharmacy, Chongqing Medical University, Chongqing, China; 10 The Department of Breast and Thyroid surgery, The First Affiliated Hospital of Chongqing Medical University, Chongqing, China; 11 The Second Clinical College of Chongqing Medical University, Chongqing, China; 12 Key Laboratory of Major Brain Disease and Aging Research (Ministry of Education), Chongqing Medical University, Chongqing, China

**Keywords:** Cancer metabolism, Breast cancer

## Abstract

Breast cancer (BC) is a common malignant tumor in women and requires a comprehensive understanding of its pathogenesis for the development of new therapeutic strategies. Polyunsaturated fatty acids (PUFAs) metabolism-driven inflammation is a causative factor in cancer development. However, the function of PUFAs′ metabolism in BC remains largely unknown. Here we report the role and underlying mechanism of epoxyoctadecenoic acids (EpOMEs), the metabolites of linoleic acid mediated by cytochrome P450 (CYP) monooxygenases, in promoting the development of BC, particularly triple-negative BC (TNBC). A metabolomics study identified that EpOMEs were significantly increased in the plasma of BC patients and MMTV-PyMT mice, which accounted for the upregulation of CYP2J2 in BC tumor tissues and tumor cells. Decreased EpOMEs by treatment of CYP monooxygenase inhibitors significantly alleviated tumor development in MMTV-PyMT mice. Treatment with EpOMEs and overexpression of *CYP2J2* to increase EpOMEs in TNBC cells significantly promoted cellular proliferation, migration, tumor growth, and metastasis. Whereas knockdown of *CYP2J2* to decrease EpOMEs inhibited tumorigenesis and lung metastasis of TNBC, which was reversed by EpOME administration. Transcriptomics and proteomics analyses revealed *CXCL9* and PLEC were critical for EpOME-mediated promotion of TNBC. Knockdown of *CXCL9* and *PLEC* inhibited TNBC progression and EpOME-mediated promotion of TNBC. Both overexpression of *CYP2J2* and EpOME treatment upregulate PLEC, while PLEC upregulates NFκB1, which is a transcription regulator of *CXCL9*. This study extends the understanding of the function of PUFAs metabolism in BC development, providing potential therapeutic targets and dietary guidelines for patients with TNBC and other BCs.

**Figure:**
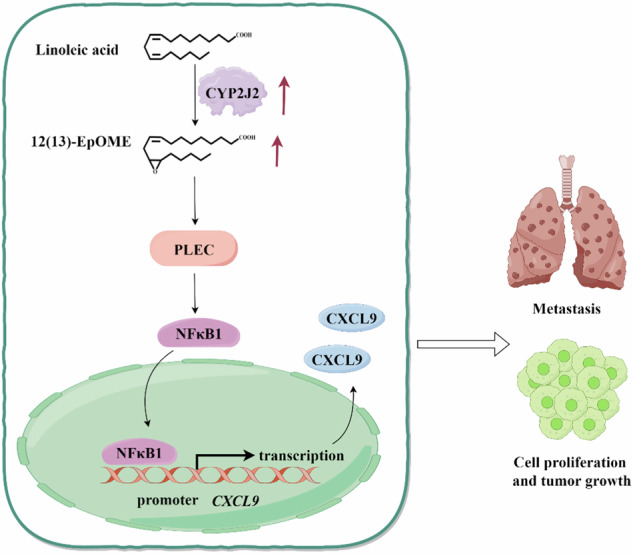
**The illustration of the hypothetical mechanism** CYP2J2/EpOMEs promotes the tumorigenesis and metastasis of TNBC via PLEC/NFKB1/CXCL9 signaling pathway.

**The illustration of the hypothetical mechanism** CYP2J2/EpOMEs promotes the tumorigenesis and metastasis of TNBC via PLEC/NFKB1/CXCL9 signaling pathway.

## Introduction

Breast cancer (BC), a major malignant tumor disease with a severe global impact on women’s health, ranks atop the incidence list of female cancers [1]. According to the latest global cancer data released by the World Health Organization, BC accounted for 2.31 million new cases in 2022, representing 11.6% of all newly diagnosed cancer cases, second only to lung cancer as the world’s second leading cancer [2]. In China, the number of new cases of BC in 2022 ranked second among women [3]. Approximately 15–20% of all breast cancer subtypes are classified as triple-negative breast cancer (TNBC) [4]. Unlike other breast cancer subtypes, TNBC is a highly heterogeneous disease, predominantly affecting younger women, and is characterized by poor differentiation, strong invasiveness, and a propensity for earlier and more frequent recurrence or metastasis, with over one-third of TNBC patients experiencing distant metastasis or relapse [5]. Recurrent or metastatic TNBC typically carries a poor prognosis, with a 5-year survival rate below 15%, markedly lower than the overall 5-year survival rate of 31% for breast cancer [6, 7]. BC not only endangers the life safety of patients but also greatly reduces the quality of life of patients because of its complicated and long treatment process and possible changes in body image [8, 9]. The inadequacy of current adjuvant therapies in ensuring optimal outcomes for all breast cancer patients underscores the challenges and necessities in breast cancer treatment, highlighting an urgent need for pathological and physio-pathological understanding of BC, particularly TNBC, to provide more precise and efficacious therapeutic strategies [10]. Therefore, multi-omics approaches, such as metabolomics, proteomics, and transcriptomics, have been extensively used to investigate the pathogenic mechanism underlying BC development [11, 12].

Recently, LC-MS/MS-based metabolomics, an effective and innovative approach to characterize the changes in metabolome, has been widely employed in the investigation of BC, including TNBC, because metabolic reprogramming is found a hallmark of cancers [13, 14]. As an important targeted metabolomics approach, metabolic profiling of the metabolism of polyunsaturated fatty acids (PUFAs) is able to simultaneously monitor the changes in the metabolites involved in three main metabolic pathways: cyclooxygenases (COXs), lipoxygenases (LOXs), and cytochrome P450s (CYPs) [15]. Many studies have shown that prostaglandins (PGs), thromboxanes (TXs), and leukotrienes (LTs), the metabolites of arachidonic acid mediated by COXs and LOXs, play important roles in the physiological and pathological processes related to cancers [16, 17]. The metabolites of PUFAs mediated by CYPs, such as epoxyeicosatrienoic acids (EETs), epoxyeicosatetraenoic acids (EpETEs) and epoxydocosapentaenoic acids (EDPs), are involved in various signal pathways in cancer and have pleiotropic functions for tumor occurrence and metastasis [18–20]. In addition, inspection of the previously reported studies, most focused on the metabolites of arachidonic acid while only a few studies reported the metabolites of other PUFAs, such as linoleic acid, eicosapentaenoic acid (EPA) and docosahexaenoic acid (DHA). While COXs- and LOXs-mediated metabolites of arachidonic acid were extensively investigated in BC [20, 21], a systemic nontargeted metabolomics of PUFAs metabolism has never been applied to the study of BC, in particular, the mechanisms and signal pathways involving CYP-mediated metabolites in the development of breast cancer remain poorly elucidated. Therefore, this study aimed to employ a targeted metabolomics of PUFAs′ metabolism to investigate the changes in the metabolites of PUFAs in three main metabolic pathways in plasma from breast cancer patients and mice for identification of the key differential metabolites and elucidation of the roles and mechanisms of these key metabolites in the development of BC/TNBC by integrating transcriptomics and proteomics, thereby furnish novel strategies for the prevention and treatment of TNBC.

## Material and methods

### Chemicals and materials

The plasmids for constructing the stable cells with overexpression and knockdown of target genes were designed and synthesized by Chengdu RabbitBio Life Technology Co (China) with the details presented in Supplementary Table S4. The antibodies, chemicals and cells used in this study were presented in Supplementary Table S5. The primers for q-PCR analysis were coined by Sangon Biotech Co (Shanghai, China) with the sequences detailed in Supplementary Table S6. The vendors of mice were described in the corresponding animal protocols in Supplementary Materials and Methods.

### Cell culture

MDA-MB-231, MCF-7, and HEK293T were grown in DMEM supplemented with 10% FBS and pen/strep. BT-474 cells were cultured with RPMI 1640 media supplemented with 10% FBS, 10 μg/mL insulin, and pen/strep. MCF-10A was grown in DMEM/F12 media supplemented with 5% Horse Serum, 20 ng/mL EGF, 0.5 μg/mL hydrocortisone, 10 μg/mL insulin, 50 ng/mL cholera and pen/strep. All cells were regularly checked for mycoplasma infection and authentication.

### Patients and specimen collection

The plasma samples of BC patients and the controls were from the Department of Clinical Laboratory of Jiangsu Cancer Hospital and Shanghai Changning Maternity and Infant Health Hospital, respectively from 2019 to 2010. The plasma used for metabolomics analysis in the present study was the remnant part after the clinical test that was separated from the fasted venous blood. Therefore, the written informed consent was approved to be waived. Thirty- three age-matched healthy controls and thirty-three pre-operative BC plasma samples were used for metabolomics analyses. The breast cancer tissue sections and age-matched surgical breast tissue sections from patients with benign breast diseases used for immunohistochemical (IHC) analysis were obtained from the Department of Pathology, the First Affiliated Hospital of Chongqing Medical University. The corresponding written informed consent was obtained from all participants.

### Targeted metabolomics analysis of the metabolites of PUFAs in plasma from clinical cohort, MMTV-PyMT mice, and control mice

The plasma levels of the metabolites of PUFAs (oxylipins) for the clinical samples were measured by a previously reported method [22]. The plasma levels of oxylipins for the MMTV-PyMT mouse model and the controls were monitored by the established targeted metabolomics method reported by Fu et al. [15].

### Proteomic analysis

MDA-MB-231 and MCF-7 were treated with medium with or without 12(13)-EpOME in three replicates, and the cells were collected, lysed to extract total proteins, and enzymatically cleaved into peptides for proteomic analysis using iTRAQ technology. The proteomic analysis protocol was performed as described in the previous study [23].

### ChIP assay

ChIP was performed using Pierce Magnetic ChIP Kit protocol (ThermoFisher Scientific, Carlsbad, CA) with NFκB1 antibody (Proteintech, China). The obtained DNA was used as a template for qPCR as described above. The primers for ChIP q-PCR analysis were coined by Sangon Biotech Co (China) with the sequences detailed in Supplementary Table S6.

### Co-IP assay

Co-IP was performed using Immunoprecipitation Kit with Protein A Magnetic Beads protocol (Beyotime, China) with PLEC antibody (Proteintech, China). The proteins obtained were detected by Western Blotting.

### Statistical analysis

Data analysis was conducted using IBM SPSS Statistics 25 (SPSS Inc., Chicago), where quantitative results are expressed as mean ± standard deviation (Mean ± SD). Statistical comparison of the two groups was performed using the Student *t*-test (normal distribution) or Wilcoxon–Mann–Whitney test (nonnormal distribution). Comparisons of means among multiple groups were determined by one-way ANOVA followed by Tukey’s or Games-Howell post-hoc comparison test. *P* values less than 0.05 are reported as statistically significant. Statistical figures were generated using GraphPad Prism version 8.0. Metabolomics and proteomics data were statistically analyzed via the online platform MetaboAnalyst 6.0, which also facilitated the creation of relevant graphical representations. Abstract graphic and schematic graphics are drawn by Figdraw.

The other experimental details are described in Supplementary Materials and Methods.

## Results

### Epoxy metabolites of linoleic acid (EpOMEs) were increased in BC patients

**Fig. 1:**
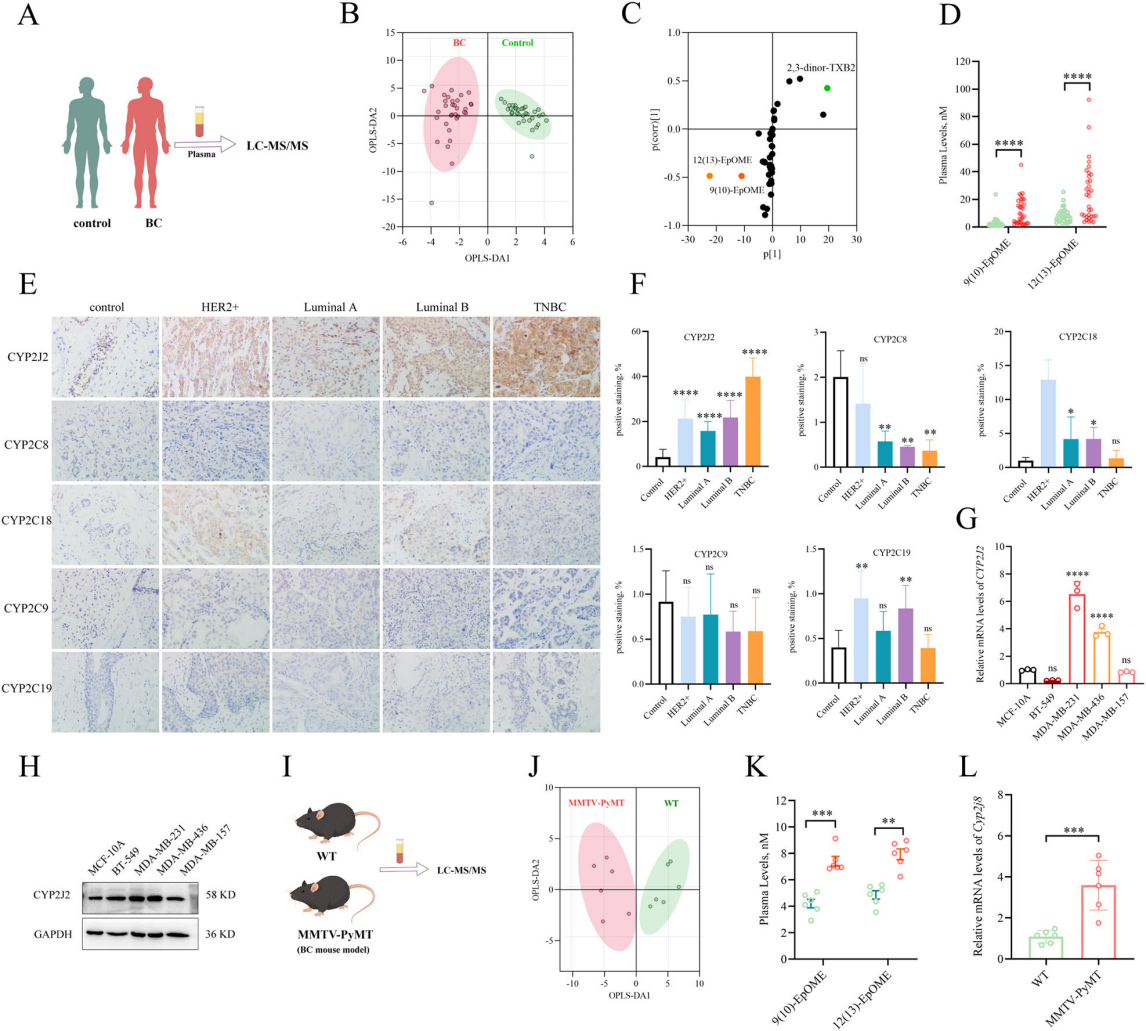
CYP2J2/EpOMEs are increased in BC patients and MMTV-PyMT mice. **A** Scheme of the clinical study; **B** OPLS-DA score scatter plots for the metabolic profile of BC patients and healthy controls (*n* = 33); **C** S-plot indicated EpOMEs are the major contributing factor to separate BC patients from controls (*n* = 33); **D** Plasma EpOMEs were significantly higher for the BC patients than those of healthy controls; Representative IHC images (**E**) and statistical analyses (**F**) of CYP2Cs and CYP2J2 expressions in tumor tissues and their para-tumoral tissues (Control) from the patients with different subtypes of BC (*n* = 10); mRNA (**G**) and protein (**H**) expression of CYP2J2 in TNBC cells and normal human mammary epithelial cells; **I** Scheme of the animal study; **J** OPLS-DA score scatter plot for metabolic profile of NTBC mice and controls (*n* = 6). **K** Plasma EpOMEs were significantly higher for MMTV-PyMT mice than those of control mice; **L** mRNA expression of *Cyp2j8* was significantly increased in the tumor tissues of MMTV-PyMT mice when compared with those of control mice. Data are presented as Mean ± SD. Statistical difference (***P < 0.001, **P < 0.01, *P < 0.05) was determined by Student *t*-test (normal distribution) or Wilcoxon–Mann–Whitney test.

To investigate the role of PUFAs′ metabolism in BC, we performed an LC-MS/MS-based targeted metabolomics to analyze the plasma levels of PUFAs′ metabolites for the preoperative BC patients (*n* = 33) and age-matched healthy controls (*n* = 33) (Fig. 1A). A total of 40 metabolites were detected, with the specific concentrations detailed in Supplementary Table S1. Orthogonal Partial Least Squares Discriminant Analysis (OPLS-DA) of these data allowed us to visually differentiate the BC group from the control group (Fig. 1B). The corresponding S-plot analysis indicated that 12(13)- and 9(10)-EpOME, as well as 2,3-dinor Thromboxane B_2_ (2,3-dinor-TXB_2_) were the major metabolites contributing to the differences between BC patients and healthy controls (Fig. 1C), which was supported by the heatmap analysis (Supplementary Fig. S1A). Since 2,3-dinor-TXB_2_ is a metabolite of TXB_2_ involved in the metabolic pathway of COXs, we selected EpOMEs, the epoxy metabolites of linoleic acid, as the target for further investigation. Further analysis revealed that both 12(13)- and 9(10)-EpOMEs were significantly elevated in plasma from the BC patients when compared with those of controls, while 12(13)-EpOME was higher than 9(10)-EpOME (Fig. 1D). In addition, the concentrations of DiHOMEs, the downstream products of EpOMEs mediated by soluble epoxide hydrolase (sEH), were slightly different between BC patients and controls (Supplementary Fig. S1C).

### Upregulated CYP monooxygenases in BC patients may contribute to the increased levels of EpOMEs

To investigate the causes of elevated plasma EpOMEs, we first examined the protein expression of their synthetic enzymes CYP2J2 and CYP2Cs in the tumor tissues of the patients with different subtypes of BCs, including HER2^+^, Luminal A, and Luminal B, as well as TNBC, and normative breast tissues from the non-tumor (Control) patients using immunohistochemical (IHC) staining. As illustrated in Fig. 1E, F, protein levels of CYP2J2 were significantly higher in tumor tissues when compared with the controls. Notably, the protein levels of CYP2J2 in tumor tissue from patients with TNBC were higher than other breast cancer subtypes. Furthermore, CYP2J2 expression was also significantly higher than the four CYP2Cs in each subtype of BC patients. In addition, we examined the expression of CYP2J2 at mRNA and protein levels for four human TNBC cell lines. We found CYP2J2 was significantly higher expressed at both mRNA and protein levels in MDA-MB-231 and MDA-MB-432 cells while was slightly higher expressed at only protein levels in BT-549 and MDA-MB-157 cells when compared with those of MCF10A cells (Fig. 1G, H). We also examined the mRNA and protein levels of CYP2J2 and 2Cs in other non-TNBC cell lines (MCF-7 and BT-474) and a human immortalized mammary epithelial cell line (MCF-10A). CYP2J2 had significantly higher expression in MCF-7 and BT-474 at both mRNA and protein levels (Supplementary Fig. S1E and S1F). The above results reiterated that CYP2J2/EpOMEs were significantly elevated in BC patients, particularly TNBC patients.

### Plasma EpOMEs and expression of *Cyp2j8* (homologous gene of human *CYP2J2*) in breast tumor were increased in MMTV-PyMT mice

Here, we used the MMTV-PyMT mouse model to study BC. H&E staining analyses showed that the tumor tissue from MMTV-PyMT mice presented the features of basal-like breast cancer, the characteristic of TNBC (Supplementary Fig. S1H). To further validate the above findings, we subsequently examined the plasma levels of the metabolites of PUFAs for the spontaneous MMTV-PyMT mice and the control mice (Fig. 1I). A total of 69 metabolites were detected in the mice plasma, which was fully presented in Supplementary Table S2. The MMTV-PyMT mice could be clearly distinguished from the control mice by OPLS-DA analysis (Fig. 1J). The plasma concentrations of 12(13)- and 9(10)-EpOMEs were significantly elevated in MMTV-PyMT mice compared with control mice (Fig. 1K, Supplementary Fig. S1B), whereas the downstream products DiHOMEs were slightly altered (Supplementary Fig. S1D). In addition, the expression of *Cyp2j8* (homologous gene of human *CYP2J2*) was significantly up-regulated, whereas the expression of *Cyp2j9*, *Cyp2c39*, and *Cyp2c40* was significantly down-regulated in the tumor tissues of MMTV-PyMT mice (Fig. 1L and Supplementary Fig. S1G). However, in the qPCR analyses, the Ct values of *Cyp2j9, Cyp2c39*, and *Cyp2c40* were about 7 higher than that of *Cyp2j8* for the same sample (Ct values: 33 vs 26), indicating the mRNA expression of *Cyp2j9, Cyp2c39*, and *Cyp2c40* were approximately 128-fold lower than that of *Cyp2j8*. Taken together, CYP2J2/EpOMEs were increased in TNBC patients, which could be further studied by using MMTV-PyMT mice model.

### Decreased EpOMEs by inhibition of CYP monooxygenases attenuated tumor development in MMTV-PyMT mice

**Fig. 2:**
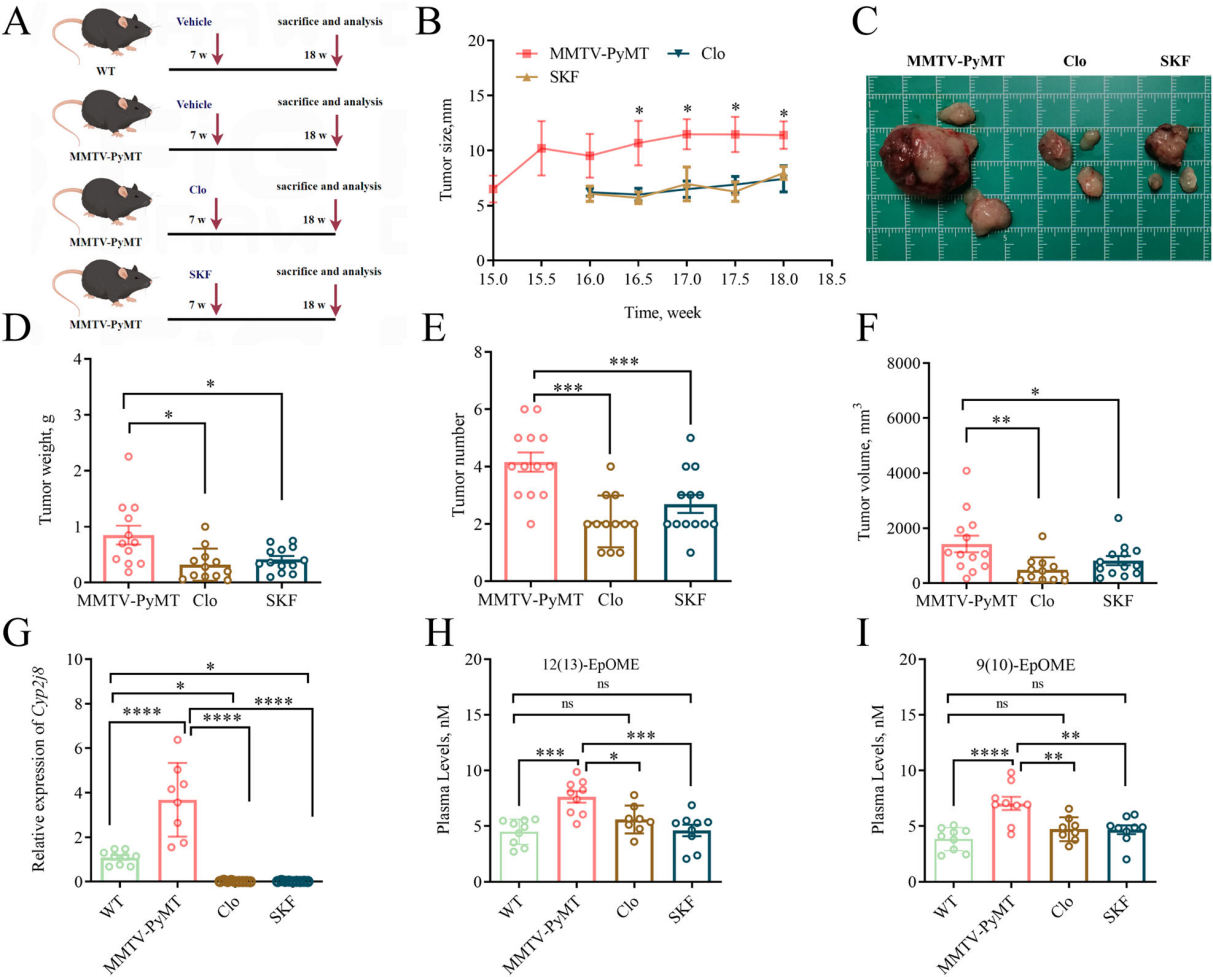
Inhibition of CYP monooxygenases alleviates BC tumor progression in mice. **A** Scheme of the animal study, here we adopted a spontaneous mice model of BC in MMTV-PyMT mice; **B** Time course of mouse tumor growth, tumor size of each mouse was measured once a week (*n* = 13); **C** Representative images of tumors collected from one mouse of the MMTV-PyMT mice treated without/with clotrimazole (Clo) or proadifen hydrochloride (SKF); Statistical analysis of the number (**D**), weight (**E**) and volume (**F**) of tumors in MMTV-PyMT mice treated without/with Clo (*n* = 12) or SKF (*n* = 13); **G** Increased mRNA expression of *Cyp2j8* in tumor tissues for MMTV-PyMT mice was significantly reduced by the treatment of Clo and SKF (*n* = 9); **H**, **I** Increased plasma levels of EpOMEs for MMTV-PyMT mice were significantly decreased by the treatment of Clo and SKF (*n* = 9). Data are presented as Mean ± SD. Statistical difference (***P < 0.001, **P < 0.01, *P < 0.05) was determined by one-way ANOVA followed by Tukey’s or Games-Howell post-hoc comparison.

To clarify the role of CYP2J2/EpOME in TNBC, we used two CYP monooxygenases inhibitors, clotrimazole (Clo) and proadifen hydrochloride (SKF), to treat MMTV-PyMT mice (Fig. 2A). We found that mammary tumors visually appeared in the MMTV-PyMT mice at the 15th week of the mice, while the tumors were visually observed in the MMTV-PyMT mice treated with here the data showed that treatment with Clo and SKF almost completely abolished the expression of Cyp2j8 at the 16th week, and the tumor sizes of the mice treated with Clo and SKF were also significantly smaller than those of the BC (Fig. 2B). As illustrated in Fig. 2C–F, the number of tumors, tumor weight, and tumor volume of the MMTV-PyMT mice treated with Clo and SKF were significantly less/lighter/smaller than those of the MMTV-PyMT mice. As expected, treatment with Clo and SKF significantly inhibited *Cyp2j8* expression in tumor tissues and reduced plasma levels of EpOMEs in MMTV-PyMT mice (Fig. 2G–I). These results indicate that inhibition of CYPs can alleviate tumor development in MMTV-PyMT mice.

### CYP2J2/EpOME promoted the proliferation of TNBC cells and TNBC tumor growth

**Fig. 3:**
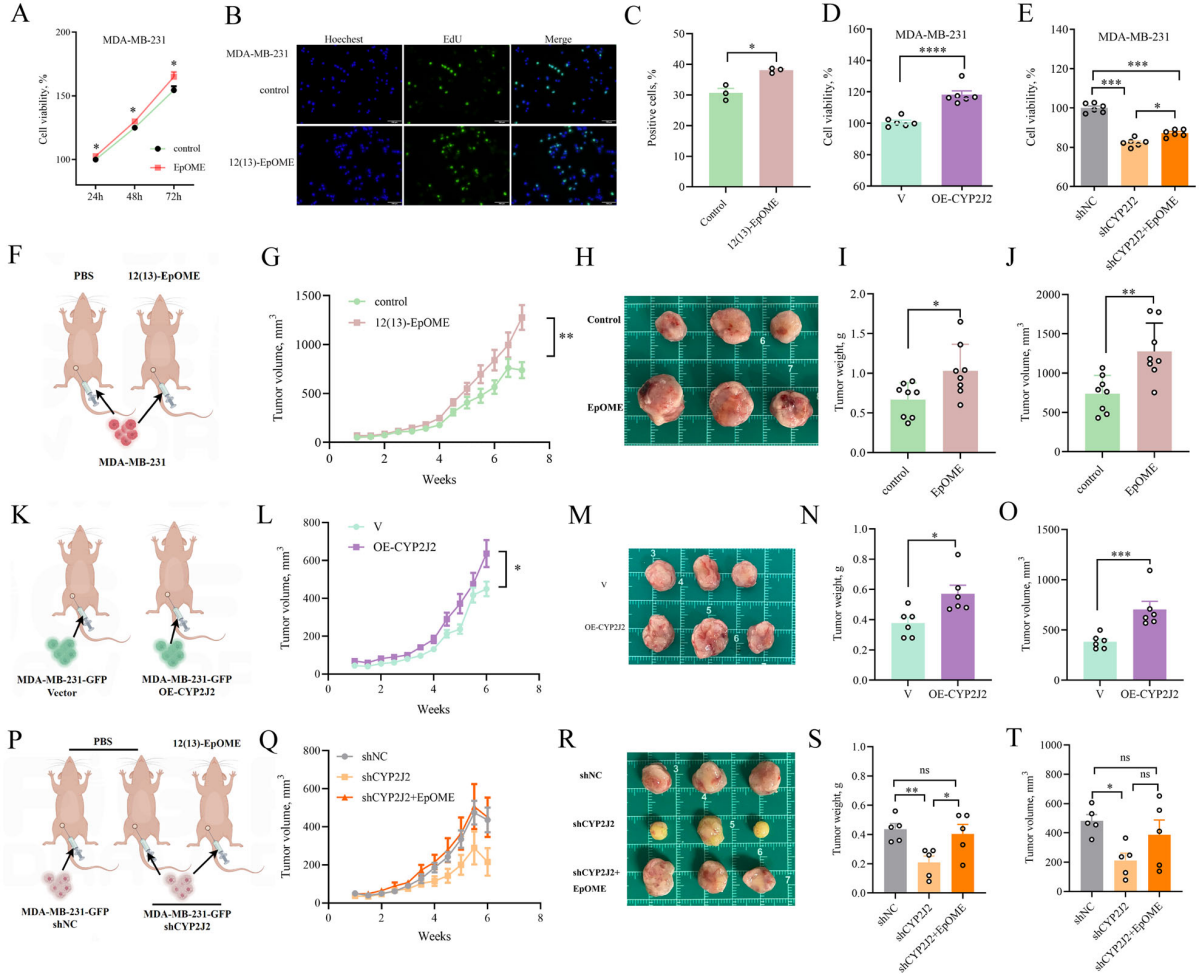
CYP2J2/EpOME promotes TNBC cellular proliferation and tumorigenesis. **A** Treatment with 12(13)-EpOME significantly increased the viability of MDA-MB-231 cells time-dependently (*n* = 3); **B**, Representative graphs of cell proliferation detected by EdU assay (scale bar: 100 μm); **C** Treatment with 12(13)-EpOME significantly increased the proliferation of MDA-MB-231 cells; **D** Overexpression (OE) of CYP2J2 significantly increased the viability of MDA-MB-231cells (*n* = 6); **E** Knockdown of *CYP2J2* (shCYP2J2) significantly decreased the viability of MDA-MB-231cells, which was counteracted by 12(13)-EpOME treatment (*n* = 6); **F** Scheme of the animal study. **G** 12(13)-EpOME treatment significantly accelerated TNBC tumorigenesis in a CDX mice model (*n* = 8); **H** Representative images of tumors from the mice treated with or without 12(13)-EpOME; Administration of 12(13)-EpOME significantly increased tumor weight (**I**) and volume (**J**) in a CDX mice model of TNBC (*n* = 8); **K** Scheme of the animal study; **L** overexpression of CYP2J2 significantly accelerated tumorigenesis in a CDX mice model of TNBC (*n* = 6); **M** Representative images of tumors from the mice inoculated with MDA-BB-231 cells with OE-CYP2J2 and control; OE-CYP2J2 significantly increased the weight (**N**) and volume (**O**) of tumors (*n* = 6) in a CDX mice model of TNBC; **P** Scheme of the animal study; **Q** Knockdown of *CYP2J2* significantly compressed tumor growth in a CDX mice model of TNBC, which was counteracted by 12(13)-EpOME treatment (*n* = 5); **R** Representative images of tumors from the mice inoculated with vehicle TNBC cells, shCYP2J2 TNBC cells, and shCYP2J2 TNBC cells+12(13)-EpOME; Knockdown of *CYP2J2* significantly decreased the tumor weight (**S**) and volume (**T**) in a CDX mice model of TNBC, which was counteracted by 12(13)-EpOME treatment. Data are presented as Mean ± SD. Statistical difference (***P < 0.001, **P < 0.01, *P < 0.05) of the two groups was determined using Student *t* -test, and comparison of multi-groups was determined using one-way ANOVA followed by Tukey’s or Games-Howell post-hoc comparison.

To investigate the roles of CYP2J2/EpOMEs in BC, especially in TNBC, we first investigated the in vitro effects of 12(13)-EpOME on MDA-MB-231 cells, the typical TNBC cells. 12(13)-EpOME was selected as the tested agent because its plasma level in BC patients and MMTV-PyMT mice was higher than those of 9(10)-EpOME. We found that treatment with 12(13)-EpOME significantly promoted the cellular viability (Fig. 3A and Supplementary Fig. S2A) time- and concentration-dependently and significantly increased the proportion of EdU-positive cells (Fig. 3B, C). We also evaluated the effects of EpOME on other TNBC cell lines. As expected, treatment of EpOME significantly increased the cellular viability and proliferation of BT-549, MDA- MB-436 and MDA-MB-157 cells. viability and proliferation (Supplementary Fig. S2J–M). These results suggest that 12(13)-EpOME promotes the viability and proliferation of TNBC cells. In addition, administration of 12(13)-EpOME significantly increased the viability and proliferation of non-TNBC cells (MCF-7 and BT-474) as well (Supplementary Fig. S2B–S2I).

To further explore the role of CYP2J2 in TNBC cells, we established cell lines with overexpression of *CYP2J2* (OE-*CYP2J2*) and knockdown of *CYP2J2* (sh*CYP2J2*) in MDA-MB-231 and MCF-7 cells via a lentiviral system (Supplementary Fig. S3A–S3D). OE-*CYP2J2* significantly increased while sh*CYP2J2* significantly decreased the production of EpOMEs (Supplementary Fig. S3E–S3F). As expected, OE-*CYP2J2* significantly increased cell viability. In contrast, sh*CYP2J2* significantly reduced cell viability, which could be reversed by the administration of 12(13)-EpOME (Fig. 3D, E and Supplementary Fig. S3G–S3H). Subsequently, we conducted in vivo studies by inoculation with MDA-MB-231 cells in a cell line-derived tumor xenograft (CDX) mice model. Both administration of 12(13)-EpOME and OE-*CYP2J2* notably accelerated primary tumor growth in mice (Fig. 3F–O). Conversely, sh*CYP2J2* markedly inhibited tumor growth, and treatment with 12(13)-EpOME effectively mitigated the sh*CYP2J2*-caused tumor-suppressive effects in vivo (Fig. 3P–T). To investigate the impact of CYP2J2/EpOME on lipid accumulation and adipocytes, we first performed hematoxylin and eosin (HE) staining on tumor tissues. No significant differences in lipid droplets or adipocytes were observed among the tumor tissues from the mice of shNC, shCYP2J2, and shCYP2J2+EpOME groups (Supplementary Fig. S3I). Also, treatment of adipocytes with EpOME resulted in insignificant changes in cell viability or lipid accumulation (Supplementary Fig. S3J, K). Furthermore, co-culture of adipocytes with MDA-MB-231 cells (treated with DMSO or EpOME) also showed insignificant changes in lipid accumulation (Supplementary Fig. S3L, M). Similarly, co-culture of adipocytes with MDA-MB-231 cells (shNC or shCYP2J2) led to insignificant changes in lipid accumulation (Supplementary Fig. S3N). In addition, qPCR analysis showed that the expression of lipid metabolism-related genes (*FASN, PPARγ, FABP4, CEBPA, SREBF1*) in adipocytes was slightly impacted by EpOME treatment (Supplementary Fig. S3O), which was supported by weak correlations between *CYP2J2* and lipid metabolism genes in basal-like breast cancer (Supplementary Fig. S3P–T) using the TIMER2.0 database. Collectively, our findings underscore the pivotal role of CYP2J2/EpOME in promoting the viability and proliferation of TNBC cells in vitro and tumor growth in vivo.

### CYP2J2/EpOME promoted migration, invasion, and lung metastasis of TNBC cells

**Fig. 4:**
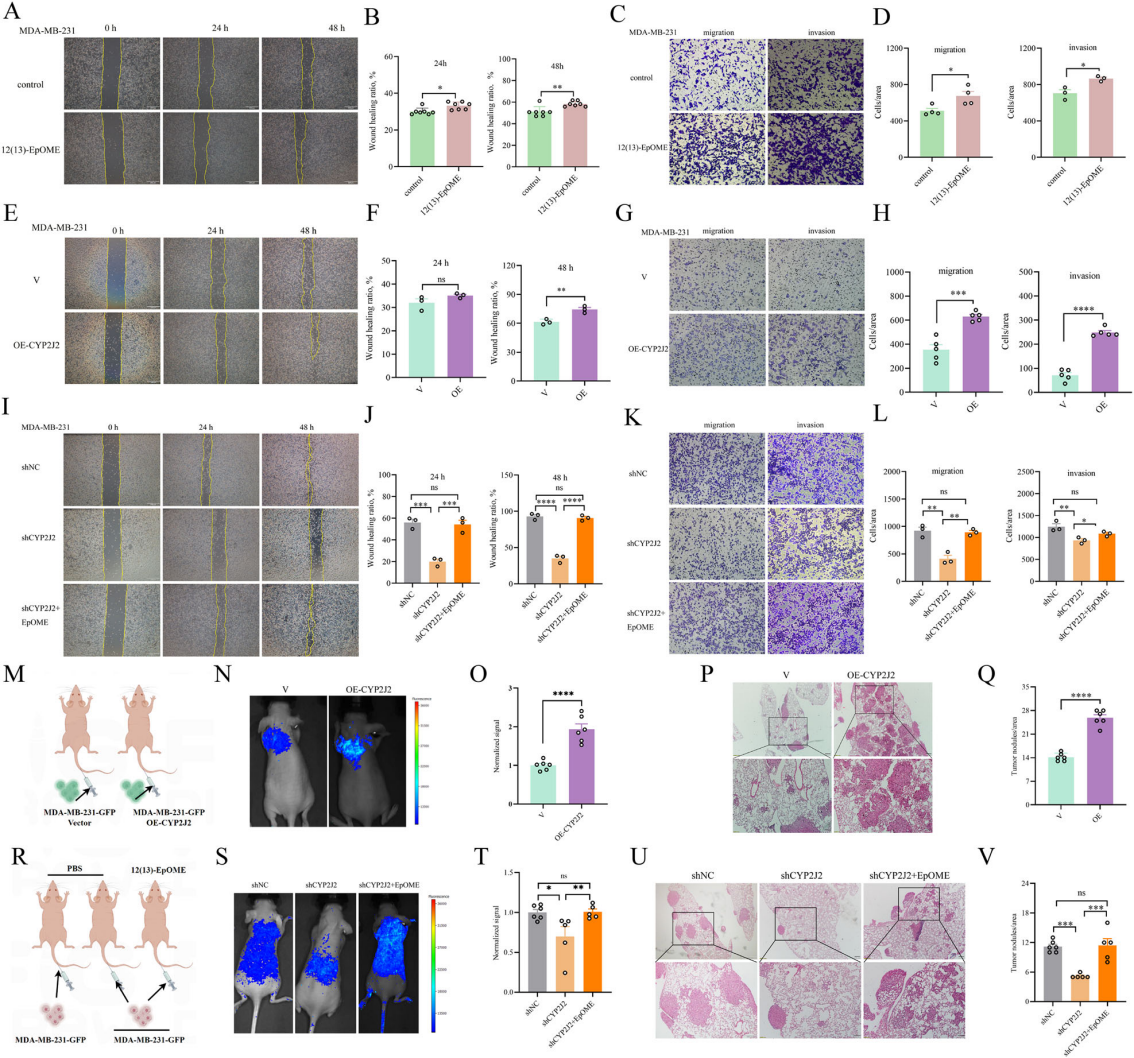
CYP2J2/EpOME promotes the invasion, migration, and metastasis of TNBC cells. **A**, **B** Administration of 12(13)-EpOME significantly accelerated wound healing capacity of MDA-MB-231 cells (*n* = 7); **C**, **D** Administration of 12(13)-EpOME significantly increased migration and invasion of MDA-MB-231 cells (*n* = 3 or 4); **E**, **F** Overexpression of CYP2J2 promoted wound healing capacity of MDA-MB-231 cells (*n* = 3); **G**, **H** Overexpression of CYP2J2 increased migration and invasion of MDA-MB-231cells (*n* = 5); **I**, **J** Knockdown of *CYP2J2* significantly compressed wound healing capacity of MDA-MB-231cells which was counteracted by 12(13)-EpOME treatment (*n* = 3); **K**, **L** Knockdown of *CYP2J2* significantly inhibited migration and invasion of MDA-MB-231 cells, which was counteracted by 12(13)-EpOME treatment; **M** Scheme of the animal study. **N**–**Q** fluorescence imaging, and histological analyses revealed overexpression of *CYP2J2* significantly increased lung metastasis of MDA-MB-231 cells (*n* = 6); **R** Scheme of the animal study; **S**–**V** fluorescence imaging, and histological analyses revealed knockdown of *CYP2J2* significantly inhibited lung metastasis of MDA-MB-231cells, which was counteracted by 12(13)-EpOME administration (*n* = 5 or 6). Data are presented as Mean ± SD. Statistical analyses were performed as Fig. 3.

Metastasis to vital organs is the predominant cause of the high mortality rate of breast cancer. To clarify the role of CYP2J2/EpOME in the process of TNBC metastasis, we tested the role of EpOME and CYP2J2 in vitro and in vivo. Administration of 12(13)-EpOME significantly increased the invasive and migratory ability of MDA-MB-231 cells (Fig. 4A–D) and other TNBC cells (BT-549, MDA-MB-436 and MDA-MB-157 cells) (Supplementary Fig. S2N–S). OE-*CYP2J2* also significantly increased the number of invasive and migratory MDA-MB-231 cells (Fig. 4E–H). In contrast, sh*CYP2J2* significantly inhibited the invasion and migration of MDA-MB-231 cells, which was abated by the treatment with 12(13)-EpOME (Fig. 4I–L). Furthermore, a CDX murine model of lung metastasis was constructed by injecting MDA-MB-231-GFP cells into Balb/c-nu mice through the tail vein. We observed mice injected with OE-*CYP2J2* cells had significantly higher metastatic colonization capacity than control cells (Fig. 4M–O), which was further supported by the histological results shown in Fig. 4P, Q. In contrast, *CYP2J2* knockdown in MDA-MB-231 cells resulted in decreased lung colonization capacity (Fig. 4R–T), and the number and area of lung metastatic nodules were also significantly reduced (Fig. 4U, V), which was effectively and significantly counteracted by 12(13)-EpOME treatment. Collectively, our results suggest that CYP2J2/EpOME promotes TNBC cell migration and invasion and mice lung metastasis.

### CYP2J2/EpOME regulated CXCL9 to promote the development of TNBC

**Fig. 5:**
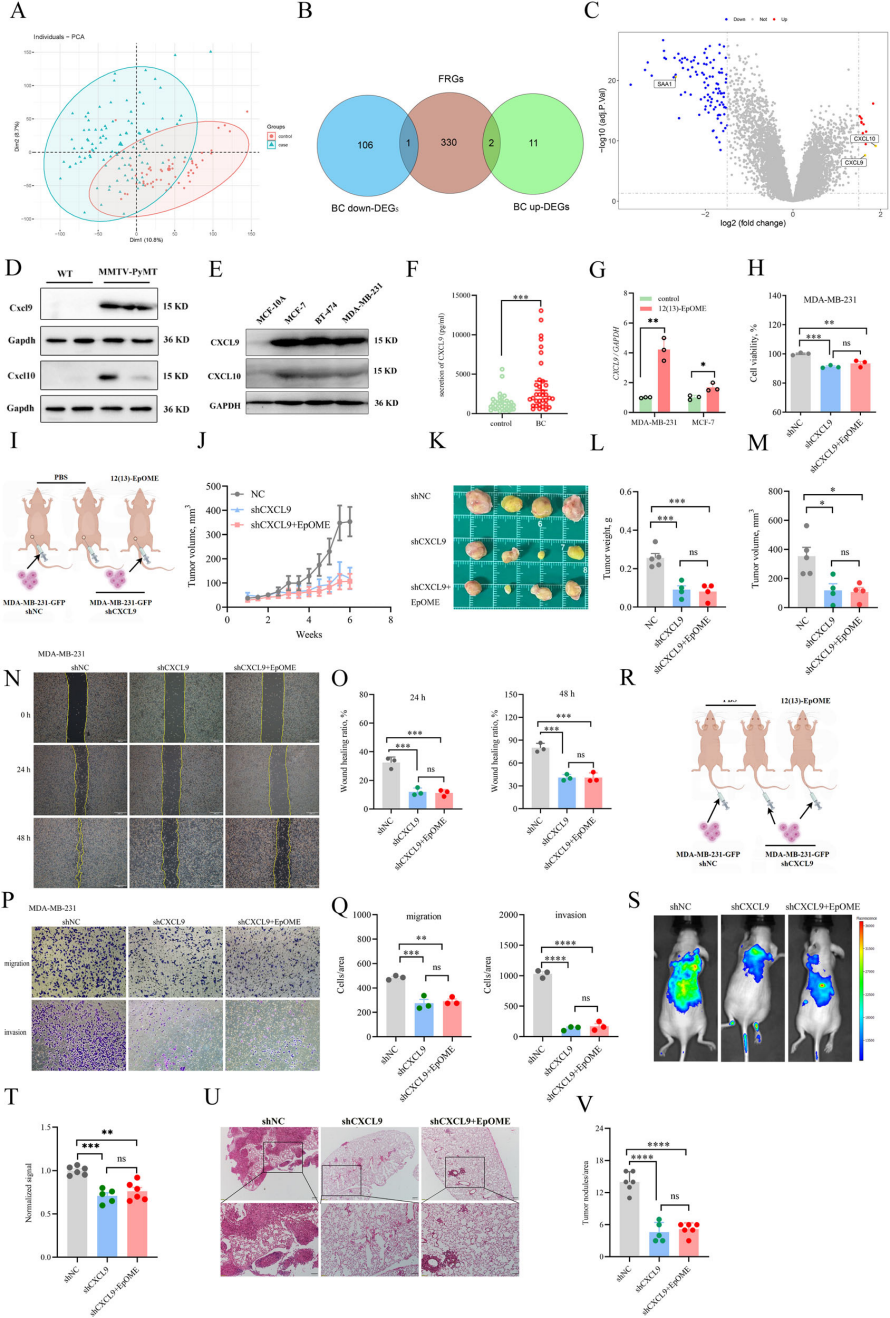
CYP2J2/EpOME regulated CXCL9 to promote the tumor growth and metastasis of TNBC. **A** PCA plots showed a visual deviation of the transcriptomes of tumor tissues from BC patients and breast tissue of controls; **B** Wein plots of DEGs versus FRGs proposed three key genes (*CXCL9*, *CXCL10* and *SAA1*) in EpOMEs promoting TNBC development; **C** a volcano plot showed transcriptomic deviation of BC patients and controls; **D** Protein Cxcl9 and Cxcl10 were increased in tumor tissues of MMTV-PyMT mice, while the increase in Cxcl9 was greater than Cxcl10; **E** Protein CXCL9 and CXCL10 was increased in TNBC, MCF-7 and BT-474 cells while the increase in CXCL9 was greater than CXCL10; **F** CXCL9 protein was significantly increased in plasma from BC patients than controls; **G** *CXCL9* were increased in MDA-MB-231 and MCF-7 cells upon 12(13)-EpOME treatment; **H** shCXCL9 decreased the cell viability of MDA-MB-231, and the cell viability-promoting effect of 12(13)-EpOME; **I** Scheme of the CDX mice model of TNBC; **J**, sh*CXCL9* inhibited the tumor growth and tumor-promoting effect of 12(13)-EpOME in a CDX mice model of TNBC; **K** Representative images of tumors from the TNBC mice with vehicle cells, and *shCXCL9* MDA-MB-231 cells receiving 12(13)-EpOME treatment and vehicle; shCXCL9 decreased the tumor weight (**L**) and volume (**M**), and the tumor-promoting effect of 12(13)-EpOME in a CDX TNBC mice model (*n* = 4 or 5); **N**, **O**, shCXCL9 decreased the wound healing capacity of MDA-MB-231 cells, and the wound healing-promoting capacity of 12(13)-EpOME (*n* = 3); **P**, **Q** shCXCL9 decreased the migrative and invasive capability of MDA-MB-231 cells, and the migration- and invasion-promoting capacity of 12(13)-EpOME (*n* = 3); **R** Scheme of the CDX mice model of TNBC metastasis; **S**, **T** fluorescence imaging showed shCXCL9decreased the lung metastasis of MDA-MB-231 cells and metastasis-promoting effect of 12(13)-EpOME (*n* = 5 or 6); **U**, **V** Histological analyses revealed shCXCL9 decreased the lung metastasis of MDA-MB-231 cells and the metastasis-promoting effect of 12(13)-EpOME (*n* = 5 or 6). Data are presented as Mean ± SD. Statistical analyses were performed as Fig. 3.

To unravel the mechanism underlying the tumor-promoting effect of CYP2J2/EpOME in TNBC, we conducted a comprehensive transcriptomics analysis involving 106 disease cohorts and 52 control cohorts (Fig. 5A). The analysis revealed a total of 107 downregulated differentially expressed genes (DEGs) and 13 upregulated DEGs (Fig. 5B). We then extended our analysis by conducting an intersection analysis, comparing the DEGs and fatty acid receptor-related genes (FRGs) obtained from the Reactome (https://reactome.org/). We ultimately identified *CXCL9*, *CXCL10*, and *SAA1* (Fig. 5B, C and Supplementary Fig. S4A), as possible target genes of EpOME. Furthermore, analyses of the GEPIA2 databases showed mRNA levels of *CXCL9* and *CXCL10* were drastically higher in BC than those of normative breast tissues (Supplementary Fig. S4B and S4C). We then examined the mRNA and protein expression of the two genes in the MMTV-PyMT tumor tissues and the control mammary tissues and found that both Cxcl9 and Cxcl10 were significantly upregulated in the tumor tissues, and significantly decreased by inhibition of CYP monooxygenases (Fig. 5D and Supplementary Fig. S4D and S4E). In addition, when compared with mammary epithelial cells, CXCL9 was highly expressed in BC cells, including MDA-MB-231, MCF-7, and BT-474 cells, and its expression was higher than CXCL10 at both mRNA and protein levels (Fig. 5E). Therefore, we selected CXCL9 for the further mechanism study. We then found the plasma levels of CXCL9 were significantly higher in the BC patients than those in the control group (Fig. 5F). Treatment of MDA-MB-231 and MCF-7 cells with 12(13)-EpOME significantly upregulated *CXCL9* expression (Fig. 5G). Furthermore, CXCL9 was significantly upregulated in OE-*CYP2J2* BC cells and decreased in sh*CYP2J2* BC cells (Supplementary Fig. S4F and S4G).

To test whether 12(13)-EpOME promotes BC cell proliferation, migration, and invasion via *CXCL9*, we established stable BC cells with knockdown of *CXCL9* for further study (Supplementary Fig. S3H). CCK-8 assay showed that knockdown of *CXCL9* significantly decreased the viability of MDA-MB-231 and MCF-7 cells and counteracted the pro-proliferative effect of 12(13)-EpOME (Fig. 5H and Supplementary Fig. S4I). A CDX mice model of tumorigenesis showed that knockdown of *CXCL9* significantly inhibited tumor growth and abrogated the tumor growth-promoting effect of 12(13)-EpOME (Fig. 5I–M). In addition, the knockdown of *CXCL9* diminished the number of invasive and migratory MDA-MB-231 cells and abolished the pro-carcinogenic effect of EpOME (Fig. 5N–P). Consistent with the in vitro assay, the knockdown of *CXCL9* resulted in TNBC cells with significantly less metastatic colonization capacity than control cells, and invalidated the metastasis-promoting effect of 12(13)-EpOME (Fig. 5R–V). Altogether, this evidence supported that *CXCL9* is the key factor for CYP2J2/EpOME to promote the development of TNBC.

### CYP2J2/EpOME upregulated PLEC to promote TNBC development

**Fig. 6:**
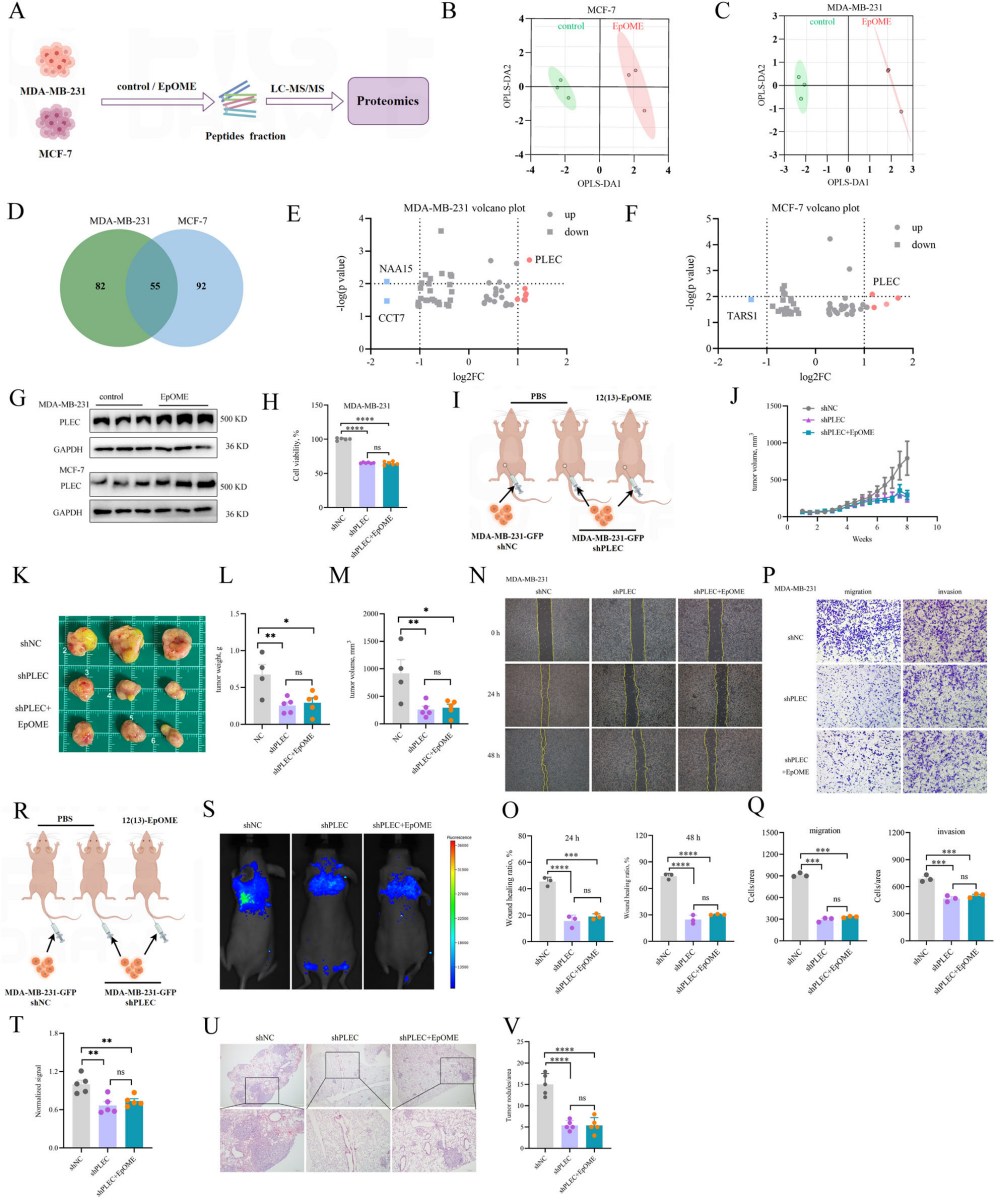
CYP2J2/EpOME mediates PLEC to promote the progression of TNBC. **A** Scheme of the proteomics experimental study; PLS-DA analysis of the proteomes showed the visual separation of MDA-MB-231 (**B**) and MCF-7 (**C**) cells upon the treatment with 12(13)-EpOME; **D** Wein diagram shown the shared DEPs of MDA-MB-231 and MCF-7 cells upon the treatment with 12(13)-EpOME; **E**, **F** Volcano plots proposed PLEC as the shared DEP contributing greatly to the separation of proteomes for MDA-MB-231 and MCF-7 cells upon the treatment with 12(13)-EpOME; **G** Treatment with 12(13)-EpOME visually upregulated PLEC in MDA-MB-231 and MCF-7 cells; **H** shPLEC significantly decreased the viability of MDA-MB-231 cells, and the cellular viability-promoting effect of 12(13)-EpOME (*n* = 6); **I** Scheme of the CDX mice model of TNBC; **J** sh*PLEC* significantly inhibited the tumor growth and tumor-promoting effect of 12(13)-EpOME in a CDX mice model of TNBC (*n* = 4 or 5); **K** Representative images of tumors from the TNBC mice with vehicle cells, and shPLEC MDA-MB-231 cells receiving 12(13)-EpOME treatment and vehicle; shCXCL9 significantly decreased the tumor weight (**L**) and tumor volume (**M**), and the tumor-promoting effect of 12(13)-EpOME in a CDX mice model of TNBC (*n* = 4 or 5); **N**, **O** shPLEC significantly decreased the wound healing capacity of MDA-MB-231 cells, and the wound healing-promoting capacity of 12(13)-EpOME (*n* = 3); **P**, **Q** shPLEC significantly decreased the migrative (left) and invasive (right) capability of MDA-MB-231 cells, and the migration-and invasion-promoting capacity of 12(13)-EpOME (*n* = 3); **R** Scheme of the CDX mice model of TNBC metastasis; **S**, **T** fluorescence imaging showed that shPLEC significantly decreased the lung metastasis of MDA-MB-231 cells and metastasis-promoting effect of 12(13)-EpOME (*n* = 5); **U**, **V** Histological analyses revealed that sh*PLEC* significantly decreased the lung metastasis of MDA-MB-231 cells and metastasis-promoting effect of 12(13)-EpOME (*n* = 5). Data are presented as Mean ± SD. Statistical analyses were performed as Fig. 3.

Besides transcriptomics, we also used proteomics to further explore the mechanisms underlying the tumor-promoting effect of CYP2J2/EpOME. We employed an iTRAQ-based proteomics analysis of MDA-MB-231 and MCF-7 cells treated with or without 12(13)-EpOME. A total of 5923 proteins were detected in MDA-MB-231 and 4658 proteins in MCF-7 cells. OPLS-DA revealed a distinct separation between the treatment and control groups (Fig. 6A–C). Differentially Expressed Proteins (DEPs) were identified between the control and treated groups according to a stringent criterion of **ǀ** fold change **ǀ** ≥ 1.2 and a *p* < 0.05. This led to the identification of 137 and 147 DEPs in MDA-MB-231 and MCF-7 cells, respectively (Supplementary Fig. S5A and S5B). Notably, 55 proteins were co-differentially expressed across both cell lines, with PLEC exhibiting the most pronounced alterations (Fig. 6D–F), which was further validated by western blotting analyses, showing that PLEC was significantly upregulated in MDA-MB-231 and MCF-7 cells upon 12(13)-EpOME treatment (Fig. 6G).

PLEC is a cytoskeletal protein with a size of 500 kD, widely expressed in mammalian tissues and cells, and plays an important role in cell tissue and signal transduction [24, 25]. IHC analyses revealed a marked upregulation of PLEC in tumor tissues from the patient with all four BC subtypes when compared to controls (Supplementary Fig. S6A, 6B). Furthermore, we observed a significant elevation in PLEC expression within MDA-MB-231 cells with overexpression of *CYP2J2*, while a significant decrease upon *CYP2J2* knockdown (Supplementary Fig. S6C).

To elucidate whether EpOME promotes breast cancer progression by mediating PLEC, we established stable MDA-MB-231 and MCF-7 cells with knockdown of *PLEC* (Supplementary Fig. S6D–S6F). Knockdown of *PLEC* markedly attenuated cellular viability and proliferation, effectively countering the proliferative boost conferred by 12(13)-EpOME treatment (Fig. 6H and Supplementary Fig. S6G). Furthermore, the knockdown of *PLEC* significantly hindered tumor growth, concurrently abrogating the tumor-boosting effect of 12(13)-EpOME (Fig. 6I, M). In addition, PLEC suppression curtailed the invasive and migratory capacities of MDA-MB-231 cells, thereby nullifying the oncogenic effects of EpOMEs (Fig. 6N–Q). Consistently, *PLEC* knockdown significantly reduced lung metastatic colonization of MDA-MB-231 cells, and abated the metastasis-promoting effect of EpOME (Fig. 6R–V).

### CYP2J2/EpOME regulated the pro-oncogenic role of CXCL9 through PLEC in TNBC

**Fig. 7:**
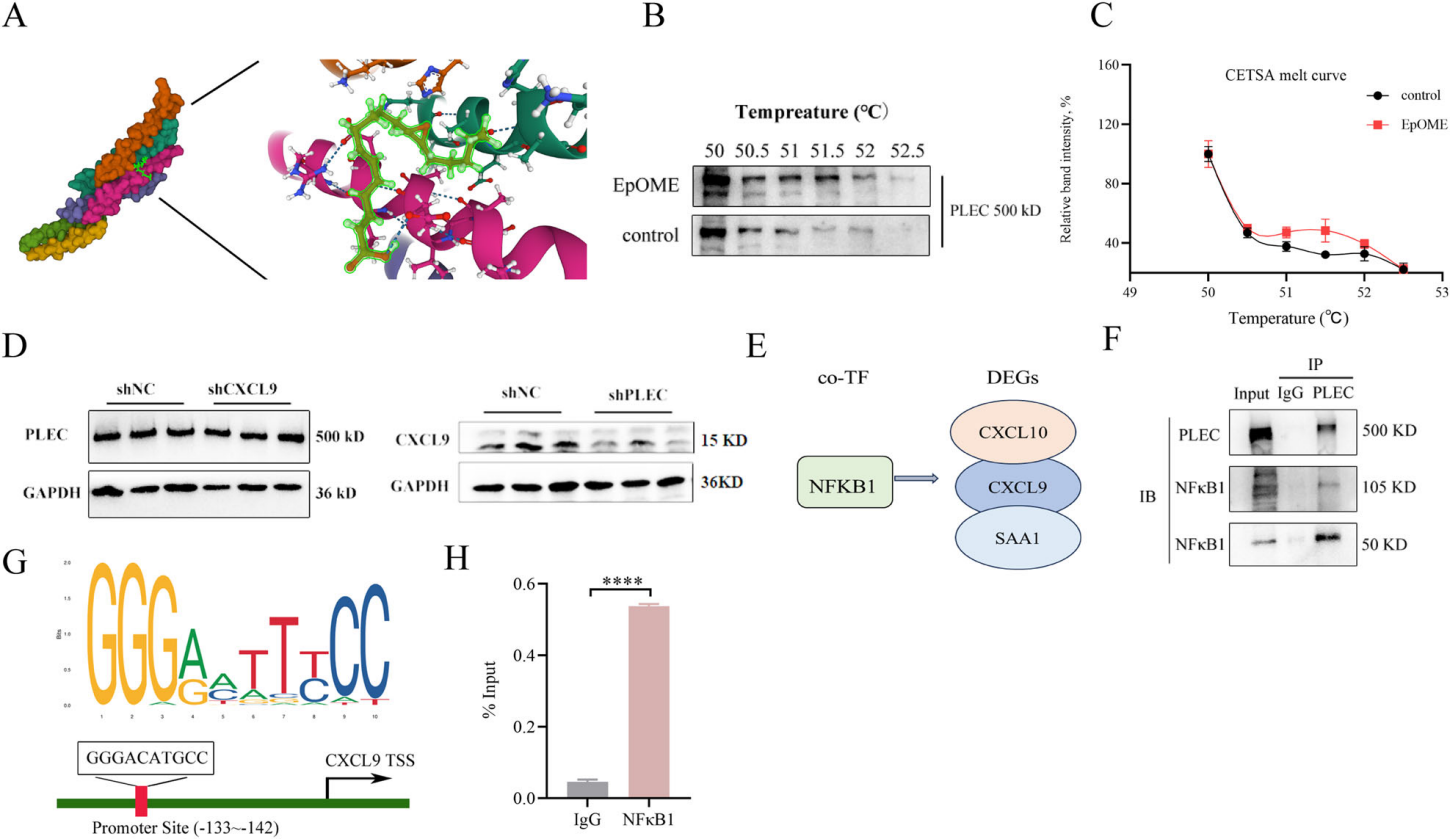
CYP2J2/EpOME regulates *CXCL9* through *PLEC* to exert pro-oncogenic effects. **A** Molecular docking predicts binding of EpOME to PLEC; **B**, **C** CETSA showed a right forward shift in thermal stability of PLEC upon the treatment of 12(13)-EpOME, indicating a direct binding of EpOME to PLEC in MDA-MB-231 cells; **D** Knockdown of *PLEC* downregulated CXCL9, but sh*CXCL9* impacts PLEC slightly; **E** NFκB1 was proposed as the transcription factor for *CXCL9*, *CXCL10*, and *SAA1* by the hTFtarget database; **F** Co-IP analysis indicated the interaction between PLEC and NFκB1; **G** NFκB1 consensus motif relative to *CXCL9* complementary region, TSS, transcription start site; **H** A ChIP assay revealed NFκB1 as the transcriptional factor of *CXCL9*. Data are presented as Mean ± SD. Statistical difference (***P < 0.001, **P < 0.01, *P < 0.05) was determined by the Student *t*-test.

To test the interaction among 12(13)-EpOME, PLEC and CXCL9 in TNBC development, we first used a molecular docking technique to reveal multiple plausive binding sites between PLEC and EpOME (Fig. 7A). We then conducted a cell thermal shift assay (CETSA) to show a rightward shift in the melting curve of PLEC post 12(13)-EpOME treatment, indicating a stabilizing effect of 12(13)-EpOME on PLEC (Fig. 7B, C). Next, we showed that the expression of CXCL9 diminished upon knockdown of *PLEC*, whereas knockdown of *CXCL9* affected PLEC expression slightly (Fig. 7D). Therefore, we deduced CXCL9 is positioned downstream of PLEC in EpOME-mediated development of TNBC.

To explore the mechanism underlying PLEC acting on CXCL9, we first proposed NFκB1 as a shared transcription factor for three DEGs, *CXCL9*, *CXCL10*, and *SAA1* in the hTFtarget database (https://guolab.wchscu.cn/hTFtarget/) (Fig. 7E). We then used the TIMER2.0 (http://timer.cistrome.org/) to reveal a positive correlation of NFκB1 with CXCL9, and PLEC as well (Supplementary Fig. S6H, S6I). The relationship between PLEC and NFκB1 was then supported by a co-immunoprecipitation (Co-IP) assay (Fig. 7F). Moreover, when PLEC was knocked down in MDA-MB-231 cells, the expression of NFκB1 was decreased accordingly (Supplementary Fig. S6J). Using JASPAR (https://jaspar2022.genereg.net/) to perform analysis of the putative *CXCL9* promoter, we discovered a powerful match to the NFκB1 transcription factor consensus motif (Fig. 7G). We then performed a Chromatin Immunoprecipitation (ChIP) assay to look for binding of NFκB1 to the promoter of *CXCL9* in MDA-MB-231 cells. ChIP qPCR showed significantly higher NFκB1 binding to the region upstream of *CXCL9* (Fig. 7H). Treatment of MDA-MB-231 cells with 12(13)-EpOME significantly upregulated NFκB1 expression (Supplementary Fig. S6J). Furthermore, NFκB1 was significantly decreased in sh*PLEC* BC cells (Supplementary Fig. S6K). Collectively, these results suggested that CYP2J2/EPOMEs play a pro-oncogenic role in TNBC by activating the PLE/NFκB1/CXCL9 signaling pathway.

## Discussion

The integrated use of omics approaches is a powerful tool to investigate pathogenic mechanisms and therapeutic targets for severe chronic diseases like cancers. This study discovered a significant increase in epoxy metabolites of linoleic acid (EpOMEs) in patients with BC by using an LC-MS/MS-based targeted metabolomics technique. The increased EpOMEs were mainly accounted for the upregulation of CYP2J2 based on the data from the clinical sample and a mice model. Then increased CYP2J2/EpOME was validated to promote tumorigenesis and metastasis of TNBC and other subtypes of BC. Subsequently, by using transcriptomics and proteomics techniques, *CXCL9* and PLEC were respectively located as the key factors for the tumor-promoting effect of EpOMEs in TNBCs. Finally, CYP2J2/EpOME was found to orchestrate CXCL9 via mediating PLEC/NFκB1 while NFκB1 transcriptionally regulated *CXCL9*.

The metabolomics study of clinical samples revealed that the major metabolites derived from COXs, LOXs, and CYPs were all increased in the plasma from BC patients, such as PGE_2_, PGD_2_ and TXB_2_, 5-HETE and 15-HETE, as well as EpOMEs and epoxyeicosatrienoic acids (EETs) (Supplementary Table S1), which is consistent with the previously reported results that COXs and LOXs are upregulated in BC patients [26–28]. Among the metabolites detected, 12(13)- and 9(10)-EpOME were identified as the major factor for BC development because the fold changes of these metabolites were significantly greater than others. Luckily, the increased plasma levels of EpOMEs were further supported by a metabolic analysis of MMTV-PyMT mice and their controls (Fig. 1K, L, Supplementary Table S1). Chocholoušková et al. utilized a metabolomics approach to reveal that 13-HODE, 9-HODE, 13-HOTrE, 9-HOTrE, and 12-HHTrE were significantly elevated in the plasma of patients with BC [29]. Both the HODEs and HOTrEs were also monitored in the clinical samples of this study with a slight difference between BC patients and healthy controls. The difference between the two studies may be accounted for by the small size of the two cohorts in the studies. Although EpOMEs were revealed for the first time to be increased in BC patients, our earlier studies found EpOMEs were significantly elevated in patients with colorectal cancer (CRC) and CRC mice [19, 30]. The awkward in previous studies is solely to reveal a role of EpOMEs in CRC development without mechanisms underlying the promoting role of EpOMEs in CRC, while the present study reported the promoting effects and underlying mechanisms of EpOMEs in the development of TNBC.

The increased EpOMEs were deduced to be mainly due to the upregulation of CYP2J2 based on the IHC analysis of the protein expression of CYP2J2/2C8/2C9/2C18/2C19 in the tumor tissues from the patients with four subtypes of BC and controls, and the mRNA and protein levels of CYP2J2/2C8/2C9/2C18/2C19 in multiple TNBC cells, other BC cells, and normative mammary cells, which was also supported by the increased *Cyp2j8* in MMTV-PyMT mice. Our finding was consistent with the previous findings that CYP2J2 is significantly highly expressed in breast cancer at mRNA levels, with the highest expression in TNBC [31]. CYP2J2 has been found to play a pro-carcinogenic role in CRC, lung cancer, and bladder cancers [32, 33]. In breast cancer, elevated expression of CYP2J2 was found associated with enhanced angiogenesis and tumor metastasis through EETs [34–36]; However, the underlying mechanism of CYP2J2/EpOMEs in TNBC remains largely unknown.

This study showed that upregulation of CYP2J2/EpOME is a cardinal driver for TNBC tumorigenesis and metastasis by knockdown and overexpression of *CYP2J2* with or without direct administration of 12(13)-EpOME in TNBC cellular models and CDX murine models of TNBC (Figs. 3 and 4). We then showed that the treatment with clotrimazole and SKF, the two structurally different non-selective CYP monooxygenase inhibitors, suppressed the tumor growth in a murine model of BC by inhibiting *Cyp2j8*/EpOMEs (Fig. 2). As illustrated in Fig. 2G, the treatment with Clo and SKF, the known CYP enzyme inhibitors, almost completely eliminated *Cyp2j8* expression, indicating that these two compounds may inhibit *Cyp2j8* by targeting at mRNA or its upstream and the used dose is sufficient for inhibiting *Cyp2j8* regardless of other Cyps. Our finding was in line with the previous studies that treatment with clotrimazole and SKF reduced viability, migration, and epithelial-mesenchymal transition (EMT) of breast cancer cells by suppressing ATP levels and glucose metabolism-related enzymes [37–39]. Altogether, the above-mentioned data highlight CYP2J2/EpOMEs as a potential therapeutic target for TNBC.

An integrated analysis of the transcriptomes of BC and fatty acid receptors from the public libraries targeted CXCL9 as a key player in EpOME-mediated TNBC development, which was further validated by its expression was manipulated by administration of EpOME and manipulation of *CYP2J2*, and its cancer-driven role in EpOME-mediated TNBC development (Fig. 5). CXCL9 is a chemokine primarily secreted by monocytes, endothelial cells, fibroblasts, and tumor cells, with pleiotropic roles across various cancers including TNBC. Recently, CXCL9 was investigated as a critical chemokine that can recruit and activate immune cells, particularly T lymphocytes, to the tumor microenvironment, thereby enhancing anti-tumor immunity in TNBC [40, 41]. In addition, CXCL9 was evident as its expression can also be hijacked by TNBC cells to promote tumor growth and metastasis [42, 43]. This study, for the first time, demonstrated that CXCL9 plays a determinant role in CYP2J2/EpOME promoting the development of TNBC by cellular models and CDX mice models of TNBC by knockdown of *CXCL9* with or without 12(13)-EpOME (Fig. 5).

An iTRAQ-based proteomics analyses of the changes in proteome of the MDA-MB-231 and MCF-7 cells upon 12(13)-EpOME treatment screened out PLEC as another key player in EpOME-mediated tumor-promoting effect because it was significantly upregulated in both cells upon 12(13)-EpOME treatment (Fig. 6E,F), which was further supported by its expression was upregulated by EpOME administration and *CYP2J2* overexpression (Fig. 6G, Supplementary Fig. S6C), and its cancer-driven role in EpOME-mediated TNBC development (Fig. 6I–V). PLEC is a cell adhesion protein to bind and stabilize membrane proteins alongside cytoskeletal components such as microtubules, actin filaments, and intermediate filaments [25, 44]. PLEC was reported previously in BC to be involved in regulating the cell cycle, thereby affecting cell proliferation and cell migration [45, 46]. Here we reported for the first time that EpOME treatment markedly enhanced TNBC cell proliferation, migration, and invasion, as well as promoting tumor growth and lung metastasis in mice via upregulation of PLEC.

Then we tried to discover the relationship between PLEC and CXCL9 in EpOME-mediated TNBC development. Is PLEC in parallel to CXCL9? Or between PLEC and CXCL9, one regulates another? PLEC was validated to positively regulate CXCL9 based on the downregulation of CXCL9 upon PLEC knockdown and slight impact of CXCL9 knockdown on PLEC expression (Fig. 7D). The binding of 12(13)-EpOME with PLEC was proposed by molecular docking (Fig. 7A), which was supported by the shift of thermo-stability of PLEC in a CETSA assay (Fig. 7B, C). Unfortunately, we tried hard but failed to get PLEC protein for further mechanism study because the molecular mass of PLEC is huge (500 kDa). In addition, a Co-IP assay revealed that PLEC could interact with NFκB1, which was validated as the transcriptional factor for *CXCL9* in TNBC cells (Fig. 7F, G). Consistently, the transcriptional regulation of *CXCL9* by NFκB1 was reported previously in Ca9-22 cells and HSC-2 cells [47]. Taken together, we unveiled that CYP2J2/EpOMEs were increased in TNBC and other subtypes of BCs, activation of which promotes TNBC development by upregulating PLEC/NFκB1/CLCL9 signaling.

One may concern that lipid accumulation play a role in the tumor-promoting effect of CYP2J2/EpOME in TNBC. However, the in vivo and in vitro results showed that neither regulation of CYP2J2 nor treatment of EpOME could significantly impact the lipid accumulation and associated genes expression in tumor tissue or adipocytes, (Fig. S3I–T), indicating that lipid accumulation plays a limited role in the tumor-promoting effect of CYP2J2/EpOME on TNBC.

It should be cautioned that EpOMEs, as the metabolites of linoleic acid (LA), could be evaluated by consumption of an LA-rich diet. A long-term intake of high LA-diet increased EpOMEs levels to exacerbate CRC in multiple mice models [19, 48]. We could speculate that the long-term consumption of a high LA diet also accelerates the development of TNBC. This study provides not only an understanding of the increased prevalence of BC in recent decades along with the lifestyle of high LA intake but also a nutrition caution for patients with TNBC.

In conclusion, our study revealed for the first time that the CYP2J2/EpOME is upregulated in TNBC, which promotes growth and metastasis of TNBC by regulating the PLEC/NFκB1/CXCL9 signaling. This study provides a novel insight into the pathogenesis of TNBC and offers potential targets and strategies for the prevention and treatment of TNBC.

## Supplementary information

Supplementary Information

Original western blots.

## Data Availability

Proteomics data are accessible at the iPROX under accession number: IPX0009462000. The datasets used and/or analyzed during the current study are available from the corresponding author upon reasonable request.
