# Supplementary material for: Epoxy metabolites of linoleic acid promote the development of breast cancer via orchestrating PLEC/NFκB1/CXCL9-mediated tumor growth and metastasis
Source: Cell Death Dis. 2024 Dec 18;15(12):901. doi: 10.1038/s41419-024-07300-6 (PMC11655665; doi:10.1038/s41419-024-07300-6)
Supplement: Supplementary file 1 — Supplementary Information [file 41419_2024_7300_MOESM1_ESM.docx]

Supplementary Information for

Epoxy Metabolites Of Linoleic Acid Promote The Development Of Breast Cancer via Orchestrating PLEC/NFκB1/CXCL9-Mediated Tumor Growth and Metastasis

Kai-Di Ni, Xian Fu, Ying Luo, Xin He, Hou-Hua Yin, Dong-Ping Mo, Jing-Xian Wu, Ming-Jun Wu, Xiao Zheng, Ya-Nan Liu, Qing Jiang, Ling-Tong Zhang, Ai-Zhi Lin, Ling Huang, Qing-Jin Pan, Xue-Dong Yin, Huan-Yu Zhang, Yi-Wen Meng, Xue Zhou, Jianbo Pan, Zufeng Guo, Jun-Yan Liu^[[1]](#footnote-1)^

The file includes:

Supplementary Materials and Methods

Supplementary Figs. S1 to S6

Supplementary Table S1 to S6

**Supplementary** **Materials and Methods**

**Cell culture protocols**

Cells were seeded into six/twelve-well plates at a density of 1×10^6^ cells per well for 24h. The cells were then treated with a corresponding medium or medium containing 12(13)-EpOME at a final concentration of 100 nM for 24 or 48 h. Then the cells were gently washed with cold PBS and harvested for the following qPCR, or western blotting experiment. The supernatant was collected for measurement of EpOME concentration as needed.

**Adipocytes isolation and co-culture**

Adipocytes isolation procedures were conducted by the method reported by Xu, Min et al.(1). In the co-culture system, mature adipocytes were cultured in the upper chamber and MDA-MB-231 cells in the lower chamber using 24-well Transwell plates. Subsequent research studies were performed after 72 hours with or without treatment.

**Cell Viability Assay of Breast Cancer Cells**

Cell viability was detected by using a CCK8 kit (TargetMol, China) following the manufacturer's protocol. In brief, cells were seeded into 96-well plates at a density of 2000 cells per well at 37 °C overnight. Subsequently, cells were cultured with media, and then 10 µL of CCK-8 solution was added to each cell of the plate. After 4 h of incubation, the plates were read at 450 nm by a microplate reader.

**EdU Assay**

Cell proliferation ability was evaluated by using a 5-ethynyl-20-deoxyuridine (EdU) assay kit (Biosharp, China) following the manufacturer’s protocol. In brief, cells were seeded into 96-well plates with a density of 3 × 10^3^ cells in each well, which were incubated with 10 μM EdU buffer at 37 °C for 3 h, fixed with 4% formaldehyde for 15 min, and permeabilized with 0.1% Triton X-100 for 15 min. EdU solution was added to the culture followed by the staining of nuclei with Hoechst. Then the results were visualized by a fluorescence microscope.

**Transwell Assay**

Migration was measured using Matrigel-free Transwell plates with an 8 μm porous membrane. In short, 2.5× 10^5^ cells were plated in the upper chambers of the Transwells. After a 24-hour incubation, migrating or invading cells were stained with 0.1% crystal violet, then photographed at 100 × and counted in four random fields. For the invasion assay, the Corning Matrigel basement membrane matrix (Corning 356234, USA) was thawed on ice at 4°C overnight. The Matrigel matrix was diluted in a serum-free medium to a final concentration of 200 μg/mL and mixed thoroughly by gently pipetting the matrix up and down. Next, 100 μL of the diluted Matrigel matrix was carefully added to the center of each Transwell insert for invasion assays. The subsequent steps are the same as in the migration assay.

**Wound Healing Assay**

Three parallel lines were drawn in advance on the bottom of a 6-well plate. Cells were placed in the plates at 5×10^5^ cells per well. After the cells reached 85–90 % confluence, a 100-µl pipette tip was used to draw a light, straight line. The floating cells in the wells were carefully washed off with PBS buffer. Images were captured under the light microscope at the 0 h, 24 h, and 48 h post-line settings.

**Quantitative Reverse Transcription-Polymerase Chain Reaction (****qRT-PCR).**

Total RNA of cells was extracted using the TRZIOL kit (Solarbio, China) according to the manufacturer’s instructions. cDNA was synthesized from 1ug total RNA using ABScript III RT Master Mix for qRT-PCR with gDNA Remover (ABclonal, China). The qRT-PCR was performed using the 2X Universal SYBR Green Fast qPCR Mix (ABclonal, China). The expression level of each gene was determined relative to *GAPDH* expression. The sequences of the mouse- and human-specific primers are listed in Supplementary Table S6.

**Oil Red O Staining**

The oil red O staining for cultured cells was performed using Oil Red O Stain Kit (Solarbio, China) according to the manufacturer’s instructions.

**H&E staining**

For H&E staining, tissues were fixed by using 4% formaldehyde, embedded in paraffin and cut. H&E staining was performed on the slices after rehydration staining with hematoxylin and eosin (Solarbio, China).

**Identification of DEGs From Transcriptome Datasets**

The series matrix file data for GSE36295 and GSE37751 were acquired from the NCBI GEO public database (https://www.ncbi.nlm.nih.gov/geo/). GSE36295 comprises 45 patients with breast cancer and 5 control individuals, while GSE37751 includes 61 patients with breast cancer and 47 control individuals. The R software packages "limma" were utilized for the analysis of these datasets, involving normalization, batch effect correction, and identification of differentially expressed genes. Specifically, the criteria for identifying DEGs were set as |log FC| ≥ 1.5 and adjusted *p*-value < 0.05. Subsequently, volcano plots were generated to visually depict the differential gene clusters. The fatty acid receptor-related genes (FRGs) were obtained from the Reactome (<https://reactome.org/>). The DEGs and FRGs were subsequently analyzed for intersection to obtain the target genes.

**Animals**

The animal experiments were conducted in accordance with the protocols approved by the Institutional Animal Care and Use Committee of the Chongqing Medical University (protocol number: IACUC-CQMU-2023-09028), which concurs with the National Institutes of Health Guidelines for the Care and Use of Laboratory Animals.

**Animal Protocol 1: A mouse model of BC**

The 6-week-old, female MMTV-PyMT transgenic mice and wild-type mice were purchased from Shanghai Model Organisms Center, Inc. The mice were randomly divided into 2 cages each holding 3 mice, and then housed in a temperature- and humidity-consistent animal room (temperature: 23 ± 1 °C; humidity: 55 ±10%) with a ventilation rate of 15 air changes per hour (ACH), a 12-hour light/dark cycle, and free access to food and water. The body weight and tumor size of each mouse were measured twice a week. The mice were anesthetized by IP injection of pentobarbital sodium (2% m/m in saline) solution with a dose of 0.1 mL/20 g body mass when they were 18 weeks old. Blood was collected through the orbital sinus into a clean Eppendorf tube containing a 10 uL antioxidant cocktail(2), which was then centrifuged at × 1500 g for 10 min to get plasma. The plasma sample then was distributed into 500-uL clean Eppendorf tubes as scheduled. The tumor and breast tissue were collected. The length (L) and width (W) of the tumors were measured with vernier calipers, and the tumor volume was calculated according to the formula V= 1/2×L×W^2^. After the length and width were measured, the mass was then recorded. All the samples were flashed frozen in liquid nitrogen and then stored under -80°C until analysis.

**Animal Protocol 2: Decreased EpOMEs by inhibition of CYP epoxygenases in a BC mice model**

Four groups were set up for this experiment, the wild mouse WT as a negative control group, the MMTV-PyMT model group, the MMTV-PyMT + CYP450 inhibitor Clotrimazole (Clo) group, and MMTV-PyMT + Proadifen hydrochloride (SKF) group. The control and model mice were fed normal chow, and the Clo and SKF groups were fed chow containing Clo and SKF, respectively. The experimental procedure was identical to that described in Protocol 1.

**Animal Protocol 3: a CDX mice model of primary tumor growth**

For the CDX mice model of the primary tumor, the breast cancer cell line, MDA-MB-231 or MDA-MB-231-GFP with overexpression or knockdown of the target gene, was treated with 12,13-EpOME or DMSO vehicle in a complete medium for 48 hours. Then, tumor cells in the exponential growth phase were harvested by brief exposure to 0.25% trypsin in 0.02% EDTA and suspended in a mixed solution of PBS and matrigel, and 5x10^6^ cells (100 μL, PBS: matrigel = 1:1) were injected into the mammary fat pad of female athymic Bablb/c-nude mice (GemPharmatech, China). Mice were subcutaneously injected with 12,13-EpOME (100 ng/mouse/day) or vehicle. The grouping and treatment information are presented in Fig. 3, 5, and 6. The body weight and tumor size of each mouse were measured twice a week. The mice were anesthetized by IP injection of pentobarbital sodium (2% m/m in saline) solution with a dose of 0.1 mL/20 g body mass 6-8 weeks after cell inoculation. Tumors were collected for picture-taking and recording the size and weight.

**Animal protocol 4: a CDX mice model of metastatic lung colonization**

Metastatic lung colonization was determined by injecting MDA-MB-231 stably expressing GFP into mice (1 × 10^5^ cells per mouse) through the tail vein. For lung colonization experiments with EpOME pretreatment, cells were treated with EpOME for 48 hours before injection. Mice were subcutaneously injected with 12,13-EpOME (100 ng/mouse/day) or vehicle. Then the fluorescence signal in vivo was measured by IVIS instrument (Clinx, China) imaging every week. At the endpoint, the lung was taken out, fixed with paraformaldehyde, and stained with hematoxylin-eosin (H&E).

**Immunohistochemistry (IHC) staining**

Paraffin-embedded sections (5 μm thickness) were prepared and dewaxed by xylene, and rehydrated by gradient ethanol solution. After being repaired by citrate buffer (Beyotime, China) antigen retrieval in a microwave for 30 min and then cooling to room temperature, the sections were washed with phosphate-buffered saline (PBS) three times, with each wash lasting 3 min. The sections were incubated with primary antibodies (Supplementary Table S5) in PBS containing 5% goat serum overnight at 4°C, then with secondary antibodies at 37°C for 1 hour. The sections were stained by using a 3,3′-diaminobenzidine (DAB) staining procedure. The sections were mounted on slides for image inspection after the nuclei were restained using a Substrate kit (Solarbio, China).

**Lentiviral packaging and infection**

The shRNA plasmid PLV-sh-zsGreen1-Puro and overexpression plasmid（CYP2J2 pCDH-GFP+PURO-3xFlag were purchased from Chengdu RabbitBio Life Technology Co (China). The shRNA sequences of target genes were listed in Supplementary Table S4. Plasmids were co-transfected with psPax2 and pMD2.G into HEK293T cells with ~70% confluency using Lipofectamine 3000 (ThermoFisher Scientific, Carlsbad, CA). GFP fluorescence was monitored for transfection efficiency. Lentiviral supernatants were harvested in 24 h and re-harvested 24 h consecutively for 1^st^ time and 2^nd^ time. Target MDA-MB-231 and MCF-7 cells were seeded in 12-well plates with ~25% confluency. Lentiviral supernatants or concentrates were added to the cells with 8 μg/mL polybrene (Beyotime, China). Transduced cells will then be selected by puromycin (Beyotime, China) for 3–6 days using a concentration based on killing curves. Gene and protein levels of target genes were analyzed by qRT-PCR and western blot, respectively for evaluation.

**Plasmid construction**

The empty eukaryotic expression vector PCDH-GFP+PURO-3xFlag was used as the expression control. CYP2J2 PCDH-GFP+PURO-3xFlag plasmid was used to construct overexpressing CYP2J2 cells.

The sequence of vector plasmid as follows: ACGCGTGTAGTCTTATGCAATACTCTTGTAGTCTTGCAACATGGTAACGATGAGTTAGCAACATGCCTTACAAGGAGAGAAAAAGCACCGTGCATGCCGATTGGTGGAAGTAAGGTGGTACGATCGTGCCTTATTAGGAAGGCAACAGACGGGTCTGACATGGATTGGACGAACCACTGAATTGCCGCATTGCAGAGATATTGTATTTAAGTGCCTAGCTCGATACAATAAACGGGTCTCTCTGGTTAGACCAGATCTGAGCCTGGGAGCTCTCTGGCTAACTAGGGAACCCACTGCTTAAGCCTCAATAAAGCTTGCCTTGAGTGCTTCAAGTAGTGTGTGCCCGTCTGTTGTGTGACTCTGGTAACTAGAGATCCCTCAGACCCTTTTAGTCAGTGTGGAAAATCTCTAGCAGTGGCGCCCGAACAGGGACCTGAAAGCGAAAGGGAAACCAGAGCTCTCTCGACGCAGGACTCGGCTTGCTGAAGCGCGCACGGCAAGAGGCGAGGGGCGGCGACTGGTGAGTACGCCAAAAATTTTGACTAGCGGAGGCTAGAAGGAGAGAGATGGGTGCGAGAGCGTCAGTATTAAGCGGGGGAGAATTAGATCGCGATGGGAAAAAATTCGGTTAAGGCCAGGGGGAAAGAAAAAATATAAATTAAAACATATAGTATGGGCAAGCAGGGAGCTAGAACGATTCGCAGTTAATCCTGGCCTGTTAGAAACATCAGAAGGCTGTAGACAAATACTGGGACAGCTACAACCATCCCTTCAGACAGGATCAGAAGAACTTAGATCATTATATAATACAGTAGCAACCCTCTATTGTGTGCATCAAAGGATAGAGATAAAAGACACCAAGGAAGCTTTAGACAAGATAGAGGAAGAGCAAAACAAAAGTAAGACCACCGCACAGCAAGCGGCCACTGATCTTCAGACCTGGAGGAGGAGATATGAGGGACAATTGGAGAAGTGAATTATATAAATATAAAGTAGTAAAAATTGAACCATTAGGAGTAGCACCCACCAAGGCAAAGAGAAGAGTGGTGCAGAGAGAAAAAAGAGCAGTGGGAATAGGAGCTTTGTTCCTTGGGTTCTTGGGAGCAGCAGGAAGCACTATGGGCGCAGCCTCAATGACGCTGACGGTACAGGCCAGACAATTATTGTCTGGTATAGTGCAGCAGCAGAACAATTTGCTGAGGGCTATTGAGGCGCAACAGCATCTGTTGCAACTCACAGTCTGGGGCATCAAGCAGCTCCAGGCAAGAATCCTGGCTGTGGAAAGATACCTAAAGGATCAACAGCTCCTGGGGATTTGGGGTTGCTCTGGAAAACTCATTTGCACCACTGCTGTGCCTTGGAATGCTAGTTGGAGTAATAAATCTCTGGAACAGATTGGAATCACACGACCTGGATGGAGTGGGACAGAGAAATTAACAATTACACAAGCTTAATACACTCCTTAATTGAAGAATCGCAAAACCAGCAAGAAAAGAATGAACAAGAATTATTGGAATTAGATAAATGGGCAAGTTTGTGGAATTGGTTTAACATAACAAATTGGCTGTGGTATATAAAATTATTCATAATGATAGTAGGAGGCTTGGTAGGTTTAAGAATAGTTTTTGCTGTACTTTCTATAGTGAATAGAGTTAGGCAGGGATATTCACCATTATCGTTTCAGACCCACCTCCCAACCCCGAGGGGACCCGACAGGCCCGAAGGAATAGAAGAAGAAGGTGGAGAGAGAGACAGAGACAGATCCATTCGATTAGTGAACGGATCTCGACGGTATCGGTTAACTTTTAAAAGAAAAGGGGGGATTGGGGGGTACAGTGCAGGGGAAAGAATAGTAGACATAATAGCAACAGACATACAAACTAAAGAATTACAAAAACAAATTACAAAATTCAAAATTTTATCGATACTAGTATTATGCCCAGTACATGACCTTATGGGACTTTCCTACTTGGCAGTACATCTACGTATTAGTCATCGCTATTACCATGGTGATGCGGTTTTGGCAGTACATCAATGGGCGTGGATAGCGGTTTGACTCACGGGGATTTCCAAGTCTCCACCCCATTGACGTCAATGGGAGTTTGTTTTGGCACCAAAATCAACGGGACTTTCCAAAATGTCGTAACAACTCCGCCCCATTGACGCAAATGGGCGGTAGGCGTGTACGGTGGGAGGTTTATATAAGCAGAGCTCGTTTAGTGAACCGTCAGATCGCCTGGAGACGCCATCCACGCTGTTTTGACCTCCATAGAAGATTCTAGAGCTAGCGAATTCGAATTTAAATCGGATCCGACTACAAAGACCATGACGGTGATTATAAAGATCATGACATCGACTACAAGGATGACGATGACAAGTAGTGAGCGGCCGCGAAGGATCTGCGATCGCTCCGGTGCCCGTCAGTGGGCAGAGCGCACATCGCCCACAGTCCCCGAGAAGTTGGGGGGAGGGGTCGGCAATTGAACGGGTGCCTAGAGAAGGTGGCGCGGGGTAAACTGGGAAAGTGATGTCGTGTACTGGCTCCGCCTTTTTCCCGAGGGTGGGGGAGAACCGTATATAAGTGCAGTAGTCGCCGTGAACGTTCTTTTTCGCAACGGGTTTGCCGCCAGAACACAGCTGAAGCTTCGAGGGGCTCGCATCTCTCCTTCACGCGCCCGCCGCCCTACCTGAGGCCGCCATCCACGCCGGTTGAGTCGCGTTCTGCCGCCTCCCGCCTGTGGTGCCTCCTGAACTGCGTCCGCCGTCTAGGTAAGTTTAAAGCTCAGGTCGAGACCGGGCCTTTGTCCGGCGCTCCCTTGGAGCCTACCTAGACTCAGCCGGCTCTCCACGCTTTGCCTGACCCTGCTTGCTCAACTCTACGTCTTTGTTTCGTTTTCTGTTCTGCGCCGTTACAGATCCAAGCTGTGACCGGCGCCTACGCTAGACGCCACCatggagagcgacgagagcggcctgcccgccatggagatcgagtgccgcatcaccggcaccctgaacggcgtggagttcgagctggtgggcggcggagagggcacccccaagcagggccgcatgaccaacaagatgaagagcaccaaaggcgccctgaccttcagcccctacctgctgagccacgtgatgggctacggcttctaccacttcggcacctaccccagcggctacgagaaccccttcctgcacgccatcaacaacggcggctacaccaacacccgcatcgagaagtacgaggacggcggcgtgctgcacgtgagcttcagctaccgctacgaggccggccgcgtgatcggcgacttcaaggtggtgggcaccggcttccccgaggacagcgtgatcttcaccgacaagatcatccgcagcaacgccaccgtggagcacctgcaccccatgggcgataacgtgctggtgggcagcttcgcccgcaccttcagcctgcgcgacggcggctactacagcttcgtggtggacagccacatgcacttcaagagcgccatccaccccagcatcctgcagaacgggggccccatgttcgccttccgccgcgtggaggagctgcacagcaacaccgagctgggcatcgtggagtaccagcacgccttcaagacccccatcgccttcgccagatcccgcgctcagtcgtccaattctgccgtggacggcaccgccggacccggctccaccggatctcgcgagggcagaggaagtcttctaacatgcggtgacgtggaggagaatcccggccctatgaccgagtacaagcccacggtgcgcctcgccacccgcgacgacgtccccagggccgtacgcaccctcgccgccgcgttcgccgactaccccgccacgcgccacaccgtcgatccggaccgccacatcgagcgggtcaccgagctgcaagaactcttcctcacgcgcgtcgggctcgacatcggcaaggtgtgggtcgcggacgacggcgccgcggtggcggtctggaccacgccggagagcgtcgaagcgggggcggtgttcgccgagatcggcccgcgcatggccgagttgagcggttcccggctggccgcgcagcaacagatggaaggcctcctggcgccgcaccggcccaaggagcccgcgtggttcctggccaccgtcggcgtctcgcccgaccaccagggcaagggtctgggcagcgccgtcgtgctccccggagtggaggcggccgagcgcgccggggtgcccgccttcctggagacctccgcgccccgcaacctccccttctacgagcggctcggcttcaccgtcaccgccgacgtcgaggtgcccgaaggaccgcgcacctggtgcatgacccgcaagcccggtgcctgaAATCAACCTCTGGATTACAAAATTTGTGAAAGATTGACTGGTATTCTTAACTATGTTGCTCCTTTTACGCTATGTGGATACGCTGCTTTAATGCCTTTGTATCATGCTATTGCTTCCCGTATGGCTTTCATTTTCTCCTCCTTGTATAAATCCTGGTTGCTGTCTCTTTATGAGGAGTTGTGGCCCGTTGTCAGGCAACGTGGCGTGGTGTGCACTGTGTTTGCTGACGCAACCCCCACTGGTTGGGGCATTGCCACCACCTGTCAGCTCCTTTCCGGGACTTTCGCTTTCCCCCTCCCTATTGCCACGGCGGAACTCATCGCCGCCTGCCTTGCCCGCTGCTGGACAGGGGCTCGGCTGTTGGGCACTGACAATTCCGTGGTGTTGTCGGGGAAATCATCGTCCTTTCCTTGGCTGCTCGCCTGTGTTGCCACCTGGATTCTGCGCGGGACGTCCTTCTGCTACGTCCCTTCGGCCCTCAATCCAGCGGACCTTCCTTCCCGCGGCCTGCTGCCGGCTCTGCGGCCTCTTCCGCGTCTTCGCCTTCGCCCTCAGACGAGTCGGATCTCCCTTTGGGCCGCCTCCCCGCCTGGTACCTTTAAGACCAATGACTTACAAGGCAGCTGTAGATCTTAGCCACTTTTTAAAAGAAAAGGGGGGACTGGAAGGGCTAATTCACTCCCAACGAAAATAAGATCTGCTTTTTGCTTGTACTGGGTCTCTCTGGTTAGACCAGATCTGAGCCTGGGAGCTCTCTGGCTAACTAGGGAACCCACTGCTTAAGCCTCAATAAAGCTTGCCTTGAGTGCTTCAAGTAGTGTGTGCCCGTCTGTTGTGTGACTCTGGTAACTAGAGATCCCTCAGACCCTTTTAGTCAGTGTGGAAAATCTCTAGCAGTAGTAGTTCATGTCATCTTATTATTCAGTATTTATAACTTGCAAAGAAATGAATATCAGAGAGTGAGAGGAACTTGTTTATTGCAGCTTATAATGGTTACAAATAAAGCAATAGCATCACAAATTTCACAAATAAAGCATTTTTTTCACTGCATTCTAGTTGTGGTTTGTCCAAACTCATCAATGTATCTTATCATGTCTGGCTCTAGCTATCCCGCCCCTAACTCCGCCCAGTTCCGCCCATTCTCCGCCCCATGGCTGACTAATTTTTTTTATTTATGCAGAGGCCGAGGCCGCCTCGGCCTCTGAGCTATTCCAGAAGTAGTGAGGAGGCTTTTTTGGAGGCCTAGACTTTTGCAGAGACGGCCCAAATTCGTAATCATGGTCATAGCTGTTTCCTGTGTGAAATTGTTATCCGCTCACAATTCCACACAACATACGAGCCGGAAGCATAAAGTGTAAAGCCTGGGGTGCCTAATGAGTGAGCTAACTCACATTAATTGCGTTGCGCTCACTGCCCGCTTTCCAGTCGGGAAACCTGTCGTGCCAGCTGCATTAATGAATCGGCCAACGCGCGGGGAGAGGCGGTTTGCGTATTGGGCGCTCTTCCGCTTCCTCGCTCACTGACTCGCTGCGCTCGGTCGTTCGGCTGCGGCGAGCGGTATCAGCTCACTCAAAGGCGGTAATACGGTTATCCACAGAATCAGGGGATAACGCAGGAAAGAACATGTGAGCAAAAGGCCAGCAAAAGGCCAGGAACCGTAAAAAGGCCGCGTTGCTGGCGTTTTTCCATAGGCTCCGCCCCCCTGACGAGCATCACAAAAATCGACGCTCAAGTCAGAGGTGGCGAAACCCGACAGGACTATAAAGATACCAGGCGTTTCCCCCTGGAAGCTCCCTCGTGCGCTCTCCTGTTCCGACCCTGCCGCTTACCGGATACCTGTCCGCCTTTCTCCCTTCGGGAAGCGTGGCGCTTTCTCATAGCTCACGCTGTAGGTATCTCAGTTCGGTGTAGGTCGTTCGCTCCAAGCTGGGCTGTGTGCACGAACCCCCCGTTCAGCCCGACCGCTGCGCCTTATCCGGTAACTATCGTCTTGAGTCCAACCCGGTAAGACACGACTTATCGCCACTGGCAGCAGCCACTGGTAACAGGATTAGCAGAGCGAGGTATGTAGGCGGTGCTACAGAGTTCTTGAAGTGGTGGCCTAACTACGGCTACACTAGAAGGACAGTATTTGGTATCTGCGCTCTGCTGAAGCCAGTTACCTTCGGAAAAAGAGTTGGTAGCTCTTGATCCGGCAAACAAACCACCGCTGGTAGCGGTGGTTTTTTTGTTTGCAAGCAGCAGATTACGCGCAGAAAAAAAGGATCTCAAGAAGATCCTTTGATCTTTTCTACGGGGTCTGACGCTCAGTGGAACGAAAACTCACGTTAAGGGATTTTGGTCATGAGATTATCAAAAAGGATCTTCACCTAGATCCTTTTAAATTAAAAATGAAGTTTTAAATCAATCTAAAGTATATATGAGTAAACTTGGTCTGACAGTTACCAATGCTTAATCAGTGAGGCACCTATCTCAGCGATCTGTCTATTTCGTTCATCCATAGTTGCCTGACTCCCCGTCGTGTAGATAACTACGATACGGGAGGGCTTACCATCTGGCCCCAGTGCTGCAATGATACCGCGAGACCCACGCTCACCGGCTCCAGATTTATCAGCAATAAACCAGCCAGCCGGAAGGGCCGAGCGCAGAAGTGGTCCTGCAACTTTATCCGCCTCCATCCAGTCTATTAATTGTTGCCGGGAAGCTAGAGTAAGTAGTTCGCCAGTTAATAGTTTGCGCAACGTTGTTGCCATTGCTACAGGCATCGTGGTGTCACGCTCGTCGTTTGGTATGGCTTCATTCAGCTCCGGTTCCCAACGATCAAGGCGAGTTACATGATCCCCCATGTTGTGCAAAAAAGCGGTTAGCTCCTTCGGTCCTCCGATCGTTGTCAGAAGTAAGTTGGCCGCAGTGTTATCACTCATGGTTATGGCAGCACTGCATAATTCTCTTACTGTCATGCCATCCGTAAGATGCTTTTCTGTGACTGGTGAGTACTCAACCAAGTCATTCTGAGAATAGTGTATGCGGCGACCGAGTTGCTCTTGCCCGGCGTCAATACGGGATAATACCGCGCCACATAGCAGAACTTTAAAAGTGCTCATCATTGGAAAACGTTCTTCGGGGCGAAAACTCTCAAGGATCTTACCGCTGTTGAGATCCAGTTCGATGTAACCCACTCGTGCACCCAACTGATCTTCAGCATCTTTTACTTTCACCAGCGTTTCTGGGTGAGCAAAAACAGGAAGGCAAAATGCCGCAAAAAAGGGAATAAGGGCGACACGGAAATGTTGAATACTCATACTCTTCCTTTTTCAATATTATTGAAGCATTTATCAGGGTTATTGTCTCATGAGCGGATACATATTTGAATGTATTTAGAAAAATAAACAAATAGGGGTTCCGCGCACATTTCCCCGAAAAGTGCCACCTGACGTCTAAGAAACCATTATTATCATGACATTAACCTATAAAAATAGGCGTATCACGAGGCCCTTTCGTCTCGCGCGTTTCGGTGATGACGGTGAAAACCTCTGACACATGCAGCTCCCGGAGACGGTCACAGCTTGTCTGTAAGCGGATGCCGGGAGCAGACAAGCCCGTCAGGGCGCGTCAGCGGGTGTTGGCGGGTGTCGGGGCTGGCTTAACTATGCGGCATCAGAGCAGATTGTACTGAGAGTGCACCATATGCGGTGTGAAATACCGCACAGATGCGTAAGGAGAAAATACCGCATCAGGCGCCATTCGCCATTCAGGCTGCGCAACTGTTGGGAAGGGCGATCGGTGCGGGCCTCTTCGCTATTACGCCAGCTGGCGAAAGGGGGATGTGCTGCAAGGCGATTAAGTTGGGTAACGCCAGGGTTTTCCCAGTCACGACGTTGTAAAACGACGGCCAGTGCCAAGCTG

The sequence of CYP2J2 PCDH-GFP+PURO-3xFlag plasmid as follows:

ACGCGTGTAGTCTTATGCAATACTCTTGTAGTCTTGCAACATGGTAACGATGAGTTAGCAACATGCCTTACAAGGAGAGAAAAAGCACCGTGCATGCCGATTGGTGGAAGTAAGGTGGTACGATCGTGCCTTATTAGGAAGGCAACAGACGGGTCTGACATGGATTGGACGAACCACTGAATTGCCGCATTGCAGAGATATTGTATTTAAGTGCCTAGCTCGATACAATAAACGGGTCTCTCTGGTTAGACCAGATCTGAGCCTGGGAGCTCTCTGGCTAACTAGGGAACCCACTGCTTAAGCCTCAATAAAGCTTGCCTTGAGTGCTTCAAGTAGTGTGTGCCCGTCTGTTGTGTGACTCTGGTAACTAGAGATCCCTCAGACCCTTTTAGTCAGTGTGGAAAATCTCTAGCAGTGGCGCCCGAACAGGGACCTGAAAGCGAAAGGGAAACCAGAGCTCTCTCGACGCAGGACTCGGCTTGCTGAAGCGCGCACGGCAAGAGGCGAGGGGCGGCGACTGGTGAGTACGCCAAAAATTTTGACTAGCGGAGGCTAGAAGGAGAGAGATGGGTGCGAGAGCGTCAGTATTAAGCGGGGGAGAATTAGATCGCGATGGGAAAAAATTCGGTTAAGGCCAGGGGGAAAGAAAAAATATAAATTAAAACATATAGTATGGGCAAGCAGGGAGCTAGAACGATTCGCAGTTAATCCTGGCCTGTTAGAAACATCAGAAGGCTGTAGACAAATACTGGGACAGCTACAACCATCCCTTCAGACAGGATCAGAAGAACTTAGATCATTATATAATACAGTAGCAACCCTCTATTGTGTGCATCAAAGGATAGAGATAAAAGACACCAAGGAAGCTTTAGACAAGATAGAGGAAGAGCAAAACAAAAGTAAGACCACCGCACAGCAAGCGGCCACTGATCTTCAGACCTGGAGGAGGAGATATGAGGGACAATTGGAGAAGTGAATTATATAAATATAAAGTAGTAAAAATTGAACCATTAGGAGTAGCACCCACCAAGGCAAAGAGAAGAGTGGTGCAGAGAGAAAAAAGAGCAGTGGGAATAGGAGCTTTGTTCCTTGGGTTCTTGGGAGCAGCAGGAAGCACTATGGGCGCAGCCTCAATGACGCTGACGGTACAGGCCAGACAATTATTGTCTGGTATAGTGCAGCAGCAGAACAATTTGCTGAGGGCTATTGAGGCGCAACAGCATCTGTTGCAACTCACAGTCTGGGGCATCAAGCAGCTCCAGGCAAGAATCCTGGCTGTGGAAAGATACCTAAAGGATCAACAGCTCCTGGGGATTTGGGGTTGCTCTGGAAAACTCATTTGCACCACTGCTGTGCCTTGGAATGCTAGTTGGAGTAATAAATCTCTGGAACAGATTGGAATCACACGACCTGGATGGAGTGGGACAGAGAAATTAACAATTACACAAGCTTAATACACTCCTTAATTGAAGAATCGCAAAACCAGCAAGAAAAGAATGAACAAGAATTATTGGAATTAGATAAATGGGCAAGTTTGTGGAATTGGTTTAACATAACAAATTGGCTGTGGTATATAAAATTATTCATAATGATAGTAGGAGGCTTGGTAGGTTTAAGAATAGTTTTTGCTGTACTTTCTATAGTGAATAGAGTTAGGCAGGGATATTCACCATTATCGTTTCAGACCCACCTCCCAACCCCGAGGGGACCCGACAGGCCCGAAGGAATAGAAGAAGAAGGTGGAGAGAGAGACAGAGACAGATCCATTCGATTAGTGAACGGATCTCGACGGTATCGGTTAACTTTTAAAAGAAAAGGGGGGATTGGGGGGTACAGTGCAGGGGAAAGAATAGTAGACATAATAGCAACAGACATACAAACTAAAGAATTACAAAAACAAATTACAAAATTCAAAATTTTATCGATACTAGTATTATGCCCAGTACATGACCTTATGGGACTTTCCTACTTGGCAGTACATCTACGTATTAGTCATCGCTATTACCATGGTGATGCGGTTTTGGCAGTACATCAATGGGCGTGGATAGCGGTTTGACTCACGGGGATTTCCAAGTCTCCACCCCATTGACGTCAATGGGAGTTTGTTTTGGCACCAAAATCAACGGGACTTTCCAAAATGTCGTAACAACTCCGCCCCATTGACGCAAATGGGCGGTAGGCGTGTACGGTGGGAGGTTTATATAAGCAGAGCTCGTTTAGTGAACCGTCAGATCGCCTGGAGACGCCATCCACGCTGTTTTGACCTCCATAGAAGATTCTAGAGCTAGCGAATTCGCCACCatgctcgcggcgatgggctctctggcggctgccctctgggcagtggtccatcctcggactctcctactgggcactgtcgcctttctgctcgctgctgactttctcaaaagacggcgcccaaagaactacccgccggggccctggcgcctgcccttccttggcaacttcttccttgtggacttcgagcagtcgcacctggaggttcagctgtttgtgaagaaatatgggaacctttttagcttggagcttggtgacatatctgcagttcttattactggcttgcccttaatcaaagaagcccttatccacatggaccaaaactttgggaaccgccccgtgacccctatgcgagaacatatctttaagaaaaatggattgattatgtcaagtggccaggcatggaaggagcaaagaaggttcactctgacagcactaaggaactttggtttaggaaagaagagcttagaggaacgcattcaggaggaggcccaacacctcactgaagcaataaaagaggagaacggacagccttttgaccctcatttcaagatcaacaatgcagtttccaatatcatttgctccatcaccttcggagaacgctttgagtaccaggatagttggtttcagcagctgctgaagttactagatgaagtcacatacttggaggcttcaaagacatgccagctctacaatgtctttccatggataatgaaattcctgcctggaccccaccaaactctcttcagcaactggaaaaaactgaaattgtttgtttctcatatgattgacaaacacagaaaggattggaatcctgcagaaacaagagactttattgatgcttaccttaaagaaatgtcaaagcacacaggcaatcctacttcaagtttccatgaagaaaacctcatctgcagcaccctggacctcttctttgccggaaccgagacaacttccacaactctgcgatgggctctgctttatatggccctctacccagaaatccaagaaaaagtacaagctgagattgacagagtgattggccaggggcagcagccgagcacagccgcccgggagtccatgccctacaccaatgctgtcatccatgaggtgcagagaatgggcaacatcatccccctgaacgttcccagggaagtgacagttgataccactttggctgggtaccacctgcccaagggtaccatgatcctgaccaatttgacggcgctgcacagggaccccacagagtgggccacccctgacacattcaatccggaccattttctggagaatggacagtttaagaaaagggaagcctttatgcctttctcaataggaaagcgggcatgcctcggagaacagttggccaggactgagctgtttattttcttcacttcccttatgcaaaaatttaccttcaggcccccaaacaatgagaagctgagcctgaagtttagaatgggtatcaccatttccccagtcagtcaccgcctctgcgctgttcctcaggtgGGATCCGACTACAAAGACCATGACGGTGATTATAAAGATCATGACATCGACTACAAGGATGACGATGACAAGTAGTGAGCGGCCGCGAAGGATCTGCGATCGCTCCGGTGCCCGTCAGTGGGCAGAGCGCACATCGCCCACAGTCCCCGAGAAGTTGGGGGGAGGGGTCGGCAATTGAACGGGTGCCTAGAGAAGGTGGCGCGGGGTAAACTGGGAAAGTGATGTCGTGTACTGGCTCCGCCTTTTTCCCGAGGGTGGGGGAGAACCGTATATAAGTGCAGTAGTCGCCGTGAACGTTCTTTTTCGCAACGGGTTTGCCGCCAGAACACAGCTGAAGCTTCGAGGGGCTCGCATCTCTCCTTCACGCGCCCGCCGCCCTACCTGAGGCCGCCATCCACGCCGGTTGAGTCGCGTTCTGCCGCCTCCCGCCTGTGGTGCCTCCTGAACTGCGTCCGCCGTCTAGGTAAGTTTAAAGCTCAGGTCGAGACCGGGCCTTTGTCCGGCGCTCCCTTGGAGCCTACCTAGACTCAGCCGGCTCTCCACGCTTTGCCTGACCCTGCTTGCTCAACTCTACGTCTTTGTTTCGTTTTCTGTTCTGCGCCGTTACAGATCCAAGCTGTGACCGGCGCCTACGCTAGACGCCACCatggagagcgacgagagcggcctgcccgccatggagatcgagtgccgcatcaccggcaccctgaacggcgtggagttcgagctggtgggcggcggagagggcacccccaagcagggccgcatgaccaacaagatgaagagcaccaaaggcgccctgaccttcagcccctacctgctgagccacgtgatgggctacggcttctaccacttcggcacctaccccagcggctacgagaaccccttcctgcacgccatcaacaacggcggctacaccaacacccgcatcgagaagtacgaggacggcggcgtgctgcacgtgagcttcagctaccgctacgaggccggccgcgtgatcggcgacttcaaggtggtgggcaccggcttccccgaggacagcgtgatcttcaccgacaagatcatccgcagcaacgccaccgtggagcacctgcaccccatgggcgataacgtgctggtgggcagcttcgcccgcaccttcagcctgcgcgacggcggctactacagcttcgtggtggacagccacatgcacttcaagagcgccatccaccccagcatcctgcagaacgggggccccatgttcgccttccgccgcgtggaggagctgcacagcaacaccgagctgggcatcgtggagtaccagcacgccttcaagacccccatcgccttcgccagatcccgcgctcagtcgtccaattctgccgtggacggcaccgccggacccggctccaccggatctcgcgagggcagaggaagtcttctaacatgcggtgacgtggaggagaatcccggccctatgaccgagtacaagcccacggtgcgcctcgccacccgcgacgacgtccccagggccgtacgcaccctcgccgccgcgttcgccgactaccccgccacgcgccacaccgtcgatccggaccgccacatcgagcgggtcaccgagctgcaagaactcttcctcacgcgcgtcgggctcgacatcggcaaggtgtgggtcgcggacgacggcgccgcggtggcggtctggaccacgccggagagcgtcgaagcgggggcggtgttcgccgagatcggcccgcgcatggccgagttgagcggttcccggctggccgcgcagcaacagatggaaggcctcctggcgccgcaccggcccaaggagcccgcgtggttcctggccaccgtcggcgtctcgcccgaccaccagggcaagggtctgggcagcgccgtcgtgctccccggagtggaggcggccgagcgcgccggggtgcccgccttcctggagacctccgcgccccgcaacctccccttctacgagcggctcggcttcaccgtcaccgccgacgtcgaggtgcccgaaggaccgcgcacctggtgcatgacccgcaagcccggtgcctgaAATCAACCTCTGGATTACAAAATTTGTGAAAGATTGACTGGTATTCTTAACTATGTTGCTCCTTTTACGCTATGTGGATACGCTGCTTTAATGCCTTTGTATCATGCTATTGCTTCCCGTATGGCTTTCATTTTCTCCTCCTTGTATAAATCCTGGTTGCTGTCTCTTTATGAGGAGTTGTGGCCCGTTGTCAGGCAACGTGGCGTGGTGTGCACTGTGTTTGCTGACGCAACCCCCACTGGTTGGGGCATTGCCACCACCTGTCAGCTCCTTTCCGGGACTTTCGCTTTCCCCCTCCCTATTGCCACGGCGGAACTCATCGCCGCCTGCCTTGCCCGCTGCTGGACAGGGGCTCGGCTGTTGGGCACTGACAATTCCGTGGTGTTGTCGGGGAAATCATCGTCCTTTCCTTGGCTGCTCGCCTGTGTTGCCACCTGGATTCTGCGCGGGACGTCCTTCTGCTACGTCCCTTCGGCCCTCAATCCAGCGGACCTTCCTTCCCGCGGCCTGCTGCCGGCTCTGCGGCCTCTTCCGCGTCTTCGCCTTCGCCCTCAGACGAGTCGGATCTCCCTTTGGGCCGCCTCCCCGCCTGGTACCTTTAAGACCAATGACTTACAAGGCAGCTGTAGATCTTAGCCACTTTTTAAAAGAAAAGGGGGGACTGGAAGGGCTAATTCACTCCCAACGAAAATAAGATCTGCTTTTTGCTTGTACTGGGTCTCTCTGGTTAGACCAGATCTGAGCCTGGGAGCTCTCTGGCTAACTAGGGAACCCACTGCTTAAGCCTCAATAAAGCTTGCCTTGAGTGCTTCAAGTAGTGTGTGCCCGTCTGTTGTGTGACTCTGGTAACTAGAGATCCCTCAGACCCTTTTAGTCAGTGTGGAAAATCTCTAGCAGTAGTAGTTCATGTCATCTTATTATTCAGTATTTATAACTTGCAAAGAAATGAATATCAGAGAGTGAGAGGAACTTGTTTATTGCAGCTTATAATGGTTACAAATAAAGCAATAGCATCACAAATTTCACAAATAAAGCATTTTTTTCACTGCATTCTAGTTGTGGTTTGTCCAAACTCATCAATGTATCTTATCATGTCTGGCTCTAGCTATCCCGCCCCTAACTCCGCCCAGTTCCGCCCATTCTCCGCCCCATGGCTGACTAATTTTTTTTATTTATGCAGAGGCCGAGGCCGCCTCGGCCTCTGAGCTATTCCAGAAGTAGTGAGGAGGCTTTTTTGGAGGCCTAGACTTTTGCAGAGACGGCCCAAATTCGTAATCATGGTCATAGCTGTTTCCTGTGTGAAATTGTTATCCGCTCACAATTCCACACAACATACGAGCCGGAAGCATAAAGTGTAAAGCCTGGGGTGCCTAATGAGTGAGCTAACTCACATTAATTGCGTTGCGCTCACTGCCCGCTTTCCAGTCGGGAAACCTGTCGTGCCAGCTGCATTAATGAATCGGCCAACGCGCGGGGAGAGGCGTTTGCGTATTGGGCGCTCTTCCGCTTCCTCGCTCACTGACTCGCTGCGCTCGGTCGTTCGGCTGCGGCGAGCGGTATCGCTCACTCAAAGGCGGTAATACGGTTATCCACAGAATCAGGGGATAACGCAGGAAAGAACATGTGAGCAAAAGGCCAGCAAAAGGCCAGGAACCGTAAAAAGGCCGCGTTGCTGGCGTTTTTCCATAGGCTCCGCCCCCCTGACGAGCATCACAAAAATCGACGCTCAAGTCAGAGGTGGCGAAACCCGACAGGACTATAAAGATACCAGGCGTTTCCCCCTGGAAGCTCCCTCGTGCGCTCTCCTGTTCCGACCCTGCCGCTTACCGGATACCTGTCCGCCTTTCTCCCTTCGGGAAGCGTGGCGCTTTCTCATAGCTCACGCTGTAGGTATCTCAGTTCGGTGTAGGTCGTTCGCTCCAAGCTGGGCTGTGTGCACGAACCCCCCGTTCAGCCCGACCGCTGCGCCTTATCCGGTAACTATCGTCTTGAGTCCAACCCGGTAAGACACGACTTATCGCCACTGGCAGCAGCCACTGGTAACAGGATTAGCAGAGCGAGGTATGTAGGCGGTGCTACAGAGTTCTTGAAGTGGTGGCCTAACTACGGCTACACTAGAAGGACAGTATTTGGTATCTGCGCTCTGCTGAAGCCAGTTACCTTCGGAAAAAGAGTTGGTAGCTCTTGATCCGGCAAACAAACCACCGCTGGTAGCGGTGGTTTTTTTGTTTGCAAGCAGCAGATTACGCGCAGAAAAAAAGGATCTCAAGAAGATCCTTTGATCTTTTCTACGGGGTCTGACGCTCAGTGGAACGAAAACTCACGTTAAGGGATTTTGGTCATGAGATTATCAAAAAGGATCTTCACCTAGATCCTTTTAAATTAAAAATGAAGTTTTAAATCAATCTAAAGTATATATGAGTAAACTTGGTCTGACAGTTACCAATGCTTAATCAGTGAGGCACCTATCTCAGCGATCTGTCTATTTCGTTCATCCATAGTTGCCTGACTCCCCGTCGTGTAGATAACTACGATACGGGAGGGCTTACCATCTGGCCCCAGTGCTGCAATGATACCGCGAGACCCACGCTCACCGGCTCCAGATTTATCAGCAATAAACCAGCCAGCCGGAAGGGCCGAGCGCAGAAGTGGTCCTGCAACTTTATCCGCCTCCATCCAGTCTATTAATTGTTGCCGGGAAGCTAGAGTAAGTAGTTCGCCAGTTAATAGTTTGCGCAACGTTGTTGCCATTGCTACAGGCATCGTGGTGTCACGCTCGTCGTTTGGTATGGCTTCATTCAGCTCCGGTTCCCAACGATCAAGGCGAGTTACATGATCCCCCATGTTGTGCAAAAAAGCGGTTAGCTCCTTCGGTCCTCCGATCGTTGTCAGAAGTAAGTTGGCCGCAGTGTTATCACTCATGGTTATGGCAGCACTGCATAATTCTCTTACTGTCATGCCATCCGTAAGATGCTTTTCTGTGACTGGTGAGTACTCAACCAAGTCATTCTGAGAATAGTGTATGCGGCGACCGAGTTGCTCTTGCCCGGCGTCAATACGGGATAATACCGCGCCACATAGCAGAACTTTAAAAGTGCTCATCATTGGAAAACGTTCTTCGGGGCGAAAACTCTCAAGGATCTTACCGCTGTTGAGATCCAGTTCGATGTAACCCACTCGTGCACCCAACTGATCTTCAGCATCTTTTACTTTCACCAGCGTTTCTGGGTGAGCAAAAACAGGAAGGCAAAATGCCGCAAAAAAGGGAATAAGGGCGACACGGAAATGTTGAATACTCATACTCTTCCTTTTTCAATATTATTGAAGCATTTATCAGGGTTATTGTCTCATGAGCGGATACATATTTGAATGTATTTAGAAAAATAAACAAATAGGGGTTCCGCGCACATTTCCCCGAAAAGTGCCACCTGACGTCTAAGAAACCATTATTATCATGACATTAACCTATAAAAATAGGCGTATCACGAGGCCCTTTCGTCTCGCGCGTTTCGGTGATGACGGTGAAAACCTCTGACACATGCAGCTCCCGGAGACGGTCACAGCTTGTCTGTAAGCGGATGCCGGGAGCAGACAAGCCCGTCAGGGCGCGTCAGCGGGTGTTGGCGGGTGTCGGGGCTGGCTTAACTATGCGGCATCAGAGCAGATTGTACTGAGAGTGCACCATATGCGGTGTGAAATACCGCACAGATGCGTAAGGAGAAAATACCGCATCAGGCGCCATTCGCCATTCAGGCTGCGCAACTGTTGGGAAGGGCGATCGGTGCGGGCCTCTTCGCTATTACGCCAGCTGGCGAAAGGGGGATGTGCTGCAAGGCGATTAAGTTGGGTAACGCCAGGGTTTTCCCAGTCACGACGTTGTAAAACGACGGCCAGTGCCAAGCTG

References

1. Xu M, Wang YM, Li WQ, Huang CL, Li J, Xie WH, et al. Ccrl2 deficiency deteriorates obesity and insulin resistance through increasing adipose tissue macrophages infiltration. Genes & diseases. 2022;9(2):429-42.

2. Liu JY, Tsai HJ, Hwang SH, Jones PD, Morisseau C, Hammock BD. Pharmacokinetic optimization of four soluble epoxide hydrolase inhibitors for use in a murine model of inflammation. Br J Pharmacol. 2009;156(2):284-96.


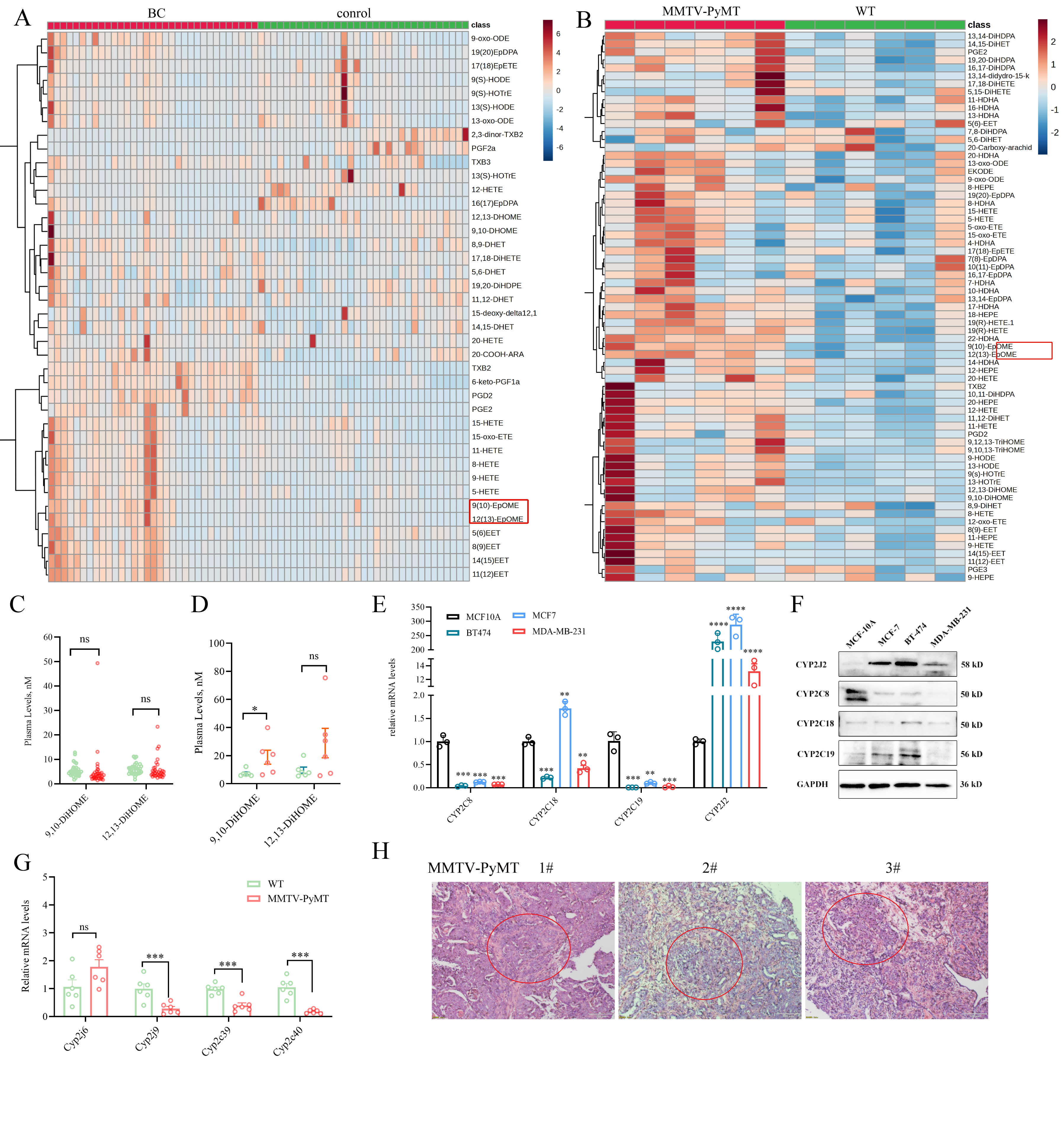


**Fig. S1.** **CYP2J2/EpOMEs are increased in BC patients, BC cells, and BC mice.**

**A,** Heatmap analysis of plasma oxylipins for BC patients and the controls (n = 33 each group); **B,** Heatmap analysis of plasma oxylipins for TNBC mice (MMTV-PyMT) and control mice (n =6 each group); **C,** Slight difference in plasma concentrations of DiHOMEs between BC patients and healthy controls (n = 33 each group); **D,** Slight difference in plasma concentrations of DiHOMEs between TNBC mice (MMTV-PyMT) and controls mice (n =6 each group); mRNA (**E**) and protein (**F**) levels of CYP2J2 were increased in human BC cells (MCF-7, BT-474, MDA-MB-231) when compared with normal human mammary epithelial cells (MCF-10A), while CYP2C8/2C18/2C19 were not. The expression CYP2C9 was too low to be detected; **G**, mRNA expression of *Cyp2cs* and *Cyp2js* in mice tumor tissues and normal mammary tissues, the mRNA expression of *Cyp2c39/2c40* and *Cyp2j6/2j9* were significantly lower than that of *Cyp2j8*. **H**, H&E staining of MMTV-PyMT tumor tissues showed features of basal-like breast cancer. Data are presented as Mean ± SD. Statistical difference (****P* < 0.001, ***P* < 0.01, **P* < 0.05) of the two groups was determined using the Student *t*-test.


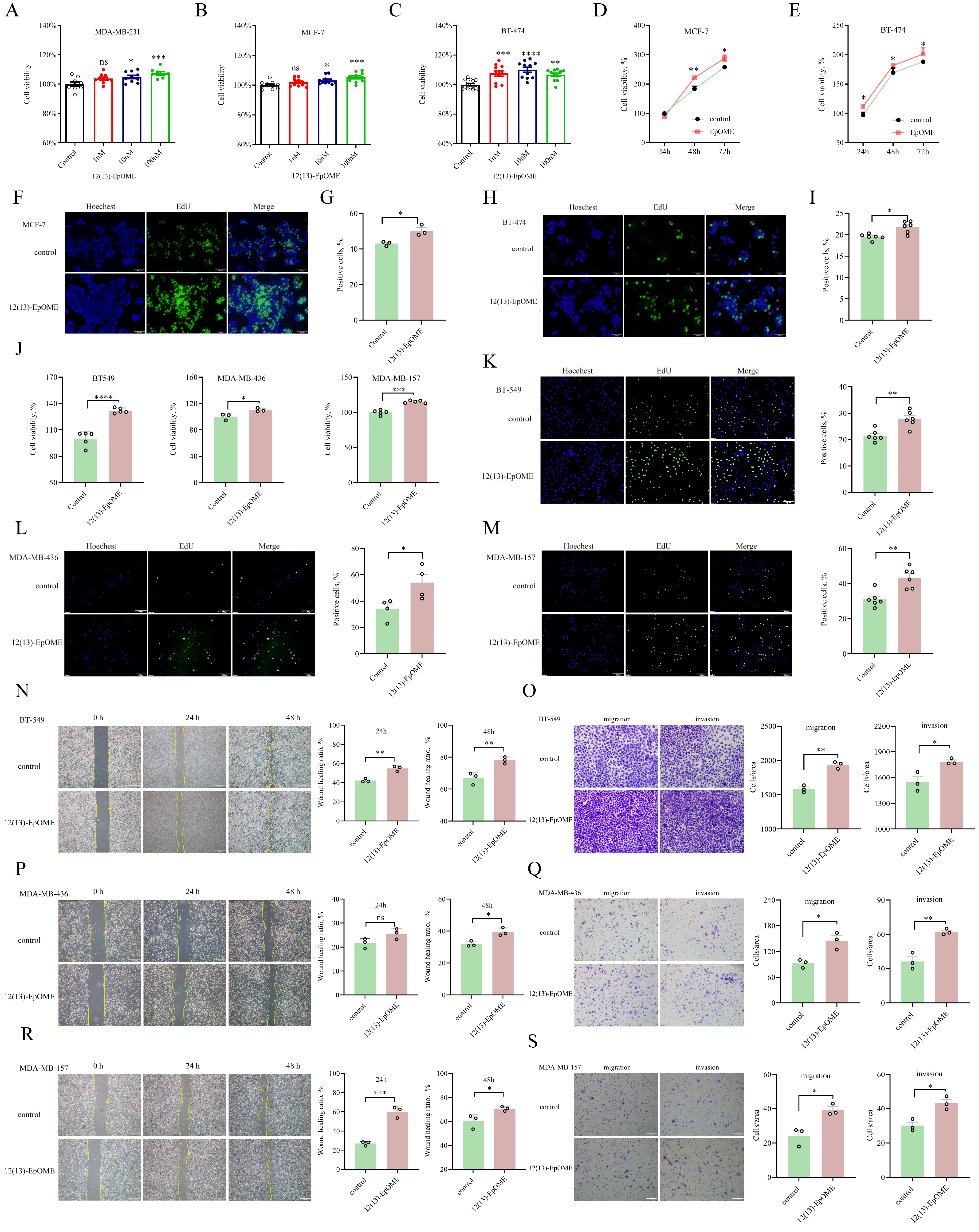


**Fig. S2. EpOME promotes the viability and proliferation of breast cancer cells *in vitro.* A**-**C**, Treatment with 12(13)-EpOME increased the viability of MDA-MB-231 cells (**A**), MCF-7 cells (**B**), and BT-474 cells (**C**) concentration-dependently (n = 9 or 12); **D** and **E**, Treatment with 12(13)-EpOME increased the viability of MCF-7 cells (**D**), and BT-474 cells (**E**) time-dependently (n = 6); **F-I**, Representative graphs of cell proliferation detected by EdU assay and statistical analysis of Edu assay for MCF-7 and BT-474 (n = 3 or 6); **J**, Treatment with 12(13)-EpOME increased the viability of three TNBC cells (BT-549, MDA-MB-436 and MDA-MB-157) (n = 3 or 5); **K-M**, Representative graphs of cell proliferation detected by EdU assay and statistical analysis of Edu assay for three TNBC cells (n = 4 or 6); N, P and R, Treatment with 12(13)-EpOME significantly accelerated wound healing capacity of three TNBC cells (n = 3); O, Q and S, Treatment with 12(13)-EpOME significantly increased migration and invasion of three TNBC cells (n = 3); Data are presented as Mean ± SD. Statistical difference (****P* < 0.001, ***P* < 0.01, **P* < 0.05) of the two groups was determined using the Student *t*-test, and comparison of multi-groups was determined using one-way ANOVA followed by Tukey's post-hoc test.


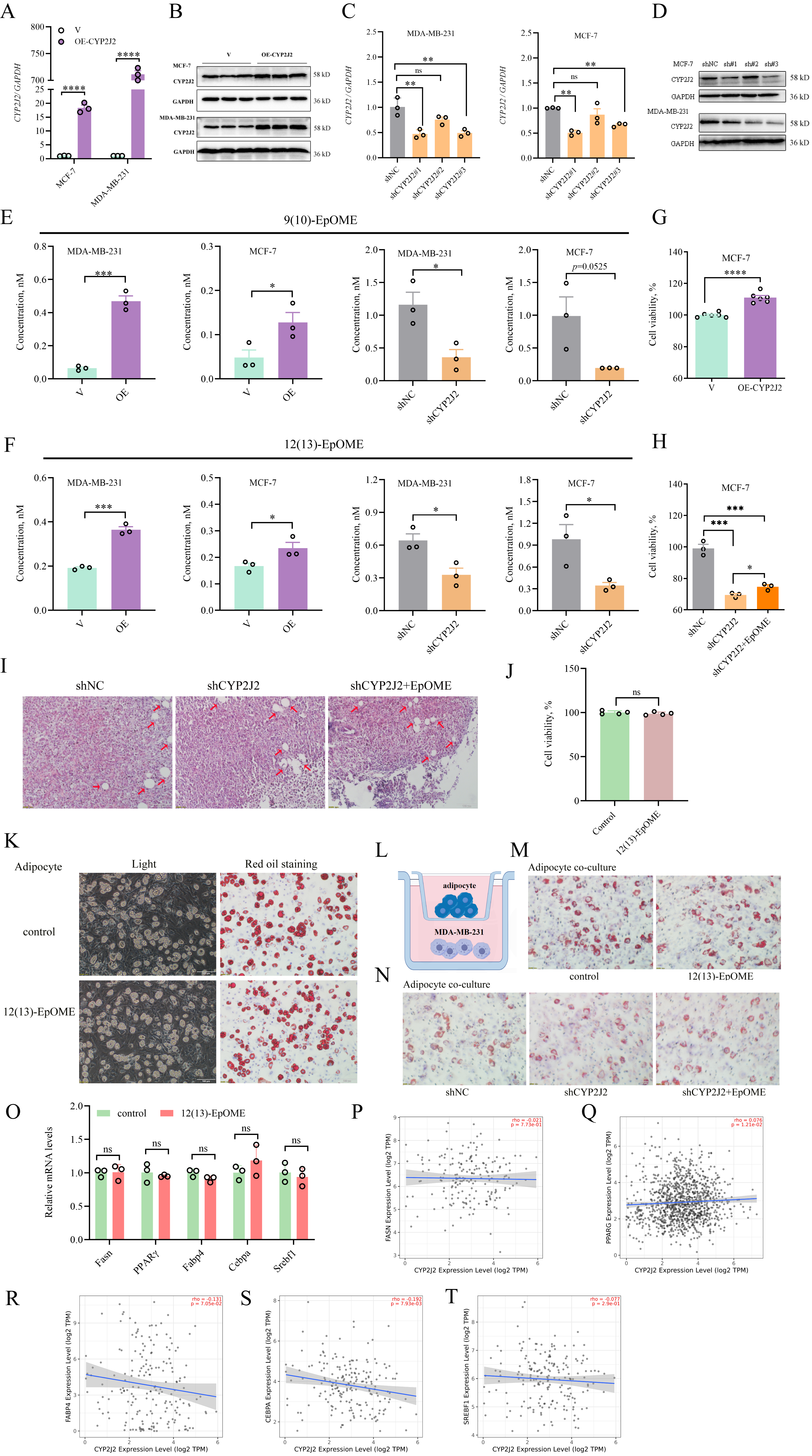


**Fig. S3. CYP2J2 promotes the viability of breast cancer cells *in vitro*. A**-**D**, Overexpression (OE) or knockdown of *CYP2J2* (*shCYP2J2*) in BC cells was evaluated by qRT-PCR and Western Blot analyses; **E** and **F**, Overexpression of *CYP2J2* significantly increased the production of EpOMEs while knockdown of *CYP2J2* significantly decreased the production of 9(10)- and 12(13)-EpOMEs (n = 3); **G,** Overexpression of *CYP2J2* significantly increased the viability of MCF-7 cells; **H**, Knockdown of *CYP2J2* significantly decreased the viability of MCF-7 cells and cellular viability-promoting effect of 12(13)-EpOME; **I**, H&E staining on tumor tissues; **J**, The CCK-8 assay showed EpOME did not significantly alter adipocyte viability (n = 4); K, Lipid accumulation in adipocytes was evaluated by oil red O staining; **L,** Scheme of co-culture of adipocytes and MDA-MB-231; **M** and **N**, Lipid accumulation in adipocytes co-culture was evaluated by oil red O staining; **O**, Determination of lipid metabolism-related gene expression by qPCR; **P-T**, Correlation between CYP2J2 and lipid metabolism genes in basal-like breast cancer analysed using the TIMER2.0 database. Data are presented as Mean ± SD. Statistical difference (****P* < 0.001, ***P* < 0.01, **P* < 0.05) of the two groups was determined using the Student *t*-test, and comparison of multi-groups was determined using one-way ANOVA followed by Tukey's post-hoc test.


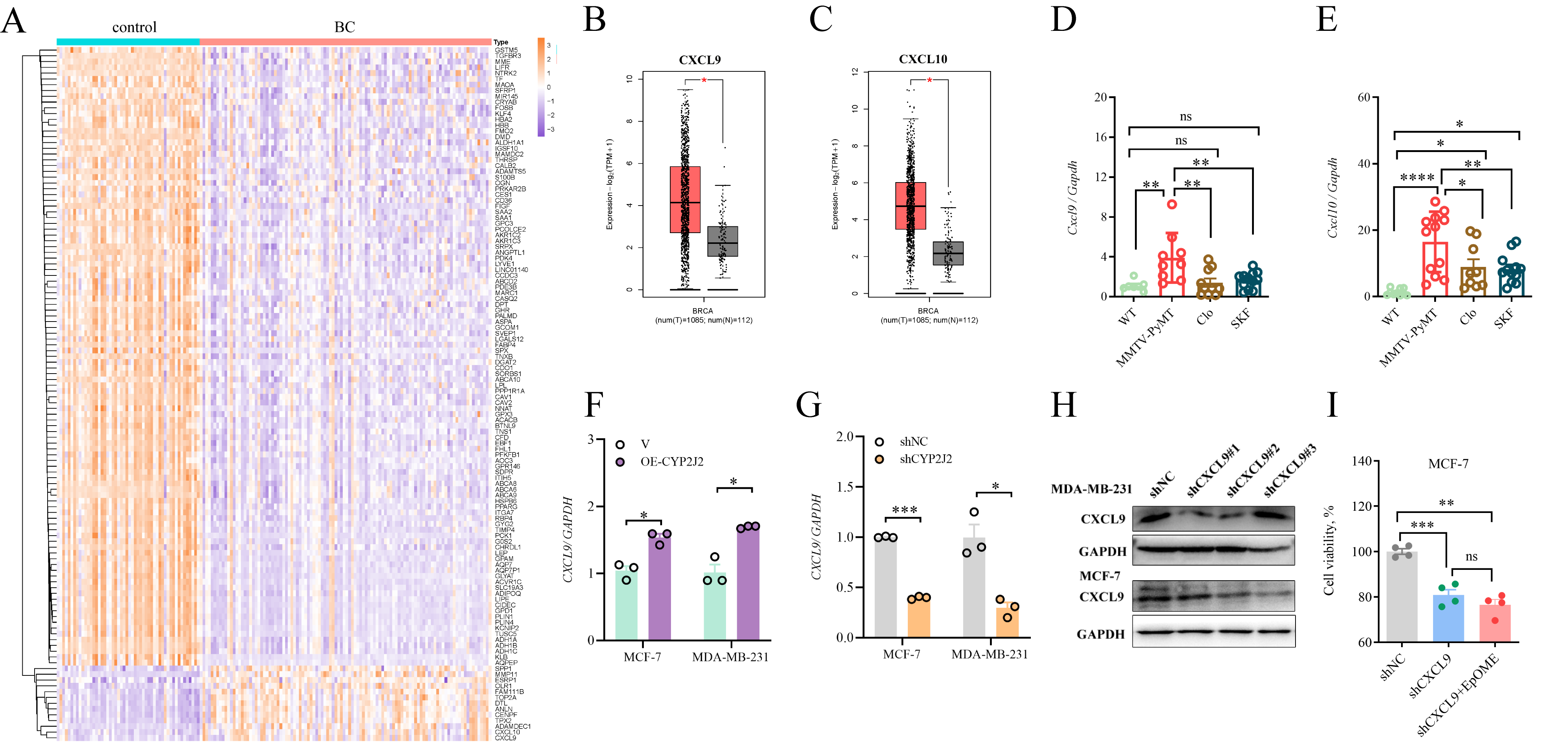


**Fig. S4. CXCL9 is associated with fatty acid metabolism in breast cancer and could be regulated by CYP2J2/EpOMEs. A**, Heatmap of DEGs between BC patients and controls; **B**, **C**, The expression levels of *CXCL9* and *CXCL10* are significantly increased in BC patients when compared with controls. Data were from the GEPIA2 database; **D**, **E**, mRNA expression levels of *Cxcl9* and *Cxcl10* were significantly increased in the tumor tissues from TNBC mice and were significantly inhibited by the treatment with Clo and SKF; **F**, **G**, OE-CYP2J2 significantly upregulated while shCYP2J2 significantly downregulated *CXCL9* in MDA-MB-231 and MCF-7 cells; **H**, Knockdown of CXCL9 (shCXCL9) was evaluated by western Blot analysis in MDA-MB-231 and MCF-7 cells; **H**, shCXCL9 significantly inhibited the cell viability of MCF-7 cells and significantly inhibited the cellular viability-promoting effect of 12(13)-EpOME. Data are presented as Mean ± SD. Statistical difference (****P* < 0.001, ***P* < 0.01, **P* < 0.05) of the two groups was determined using the Student *t*-test, and comparison of multi-groups was determined using one-way ANOVA followed by Tukey's post-hoc test.

**
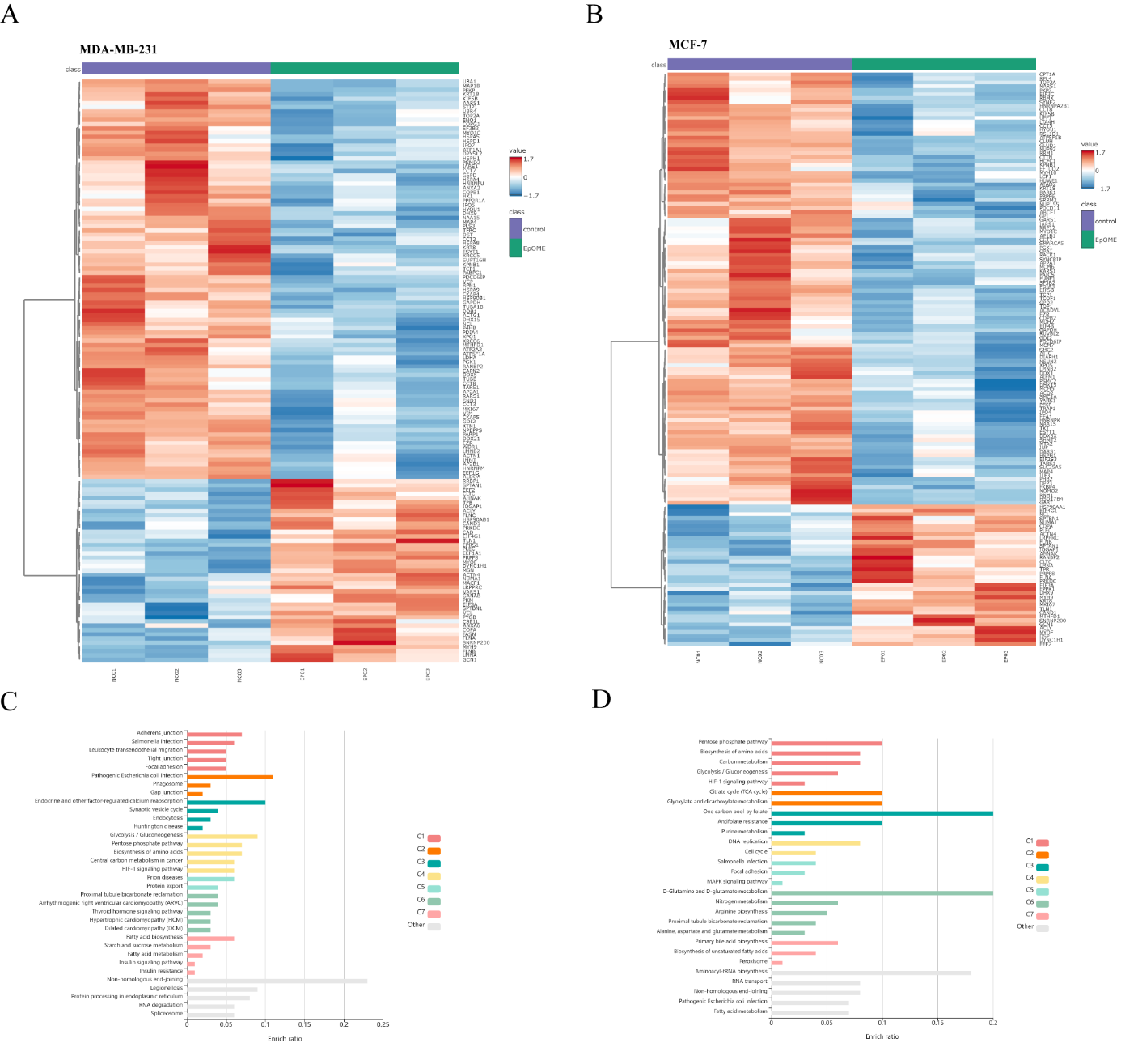
**

**Fig. S5. Proteomics analyses of the downstream target proteins of EpOME. A**, Heatmap analyses of DEPs in the proteomes of MDA-MB-231 cells upon the treatment of 12(13)-EpOME; **B**, Heatmap analyses of DEPs in the proteomes of MCF-7 cells upon the treatment of 12(13)-EpOME; **C**, **D**, KEGG Enrichment_heatmap of DEPs in the proteomes of MDA-MB-231 cells upon the treatment of 12(13)-EpOME; **D**, KEGG Enrichment_heatmap of DEPs in the proteomes of MCF-7 cells upon the treatment of 12(13)-EpOME.


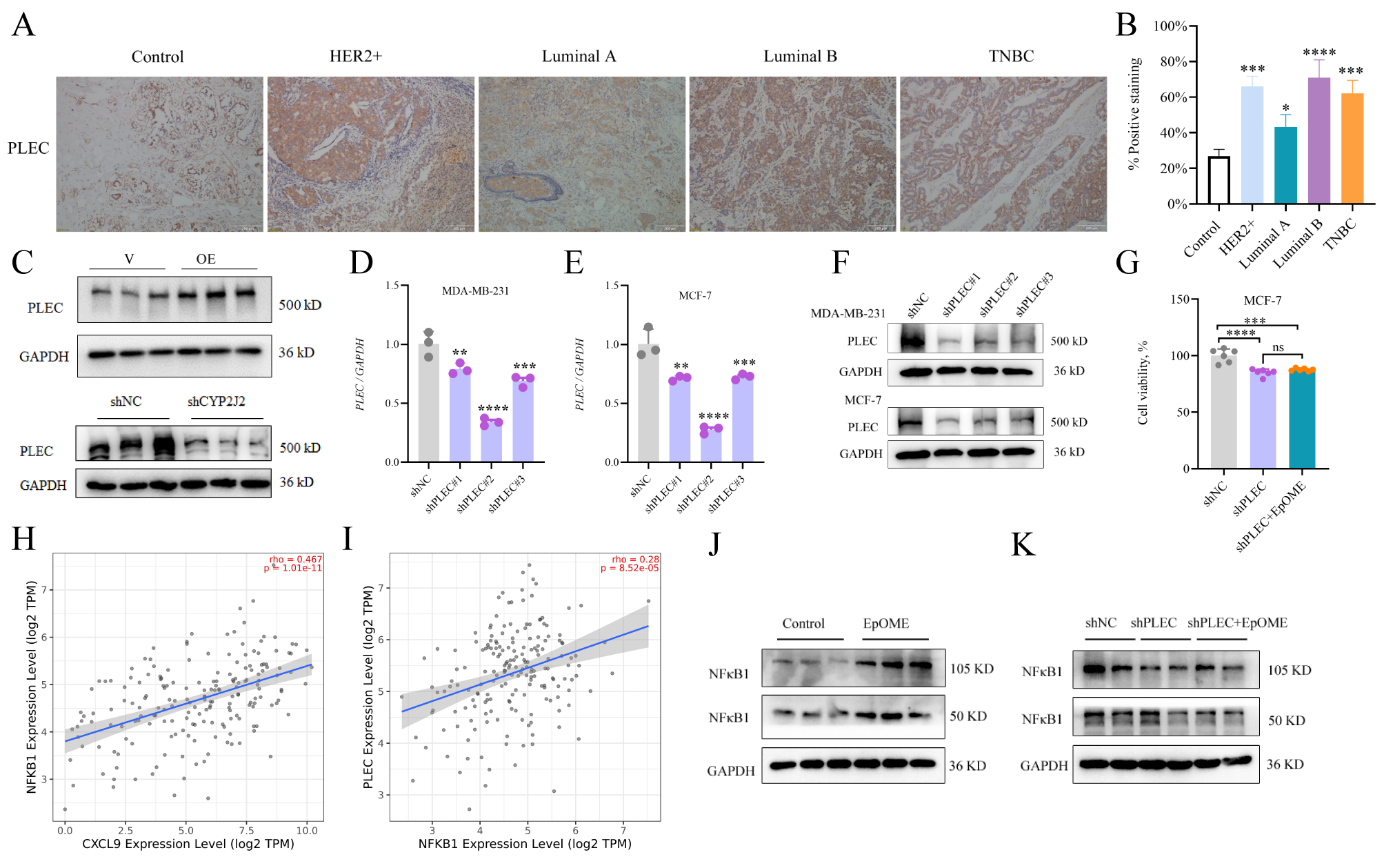


**Fig. S6. CYP2J2/EpOME promotes the progression of breast cancer by regulating PLEC.**

**A**, Representative IHC images of PLEC expression in tumor tissues from four subtypes of BC patients and the normative mammary breast tissues from controls; **B**, PLEC expression was significantly increased in tumor tissues from four subtypes of BC patients when compared with those of normal mammary tissues (n =10 per group). **C**, OE-CYP2J2 upregulated PLEC while shCYP2J2 downregulated PLEC in MDA-MB-231 cells; **D-F**, Knockdown of *PLEC* in MDA-MB-231 and MCF-7 cells was evaluated by qRT-PCR and WB analyses; **G**, shPLEC significantly inhibited the cell viability of MCF-7 cells and significantly inhibited the cellular viability-promoting effect of 12(13)-EpOME; **H** and **I**, NFκB1 is positively correlated with CXCL9 (H) and PLEC (I) in the tumor tissues from TNBC patients by Pearson's correlation analysis. **J**, NFκB1 was upregulated by 12(13)-EpOME in MDA-MB-231; **K**, NFκB1 was downregulated in MDA-MB-231 by knockdown of PLEC. Data are presented as Mean ± SD. Statistical difference (****P* < 0.001, ***P* < 0.01, **P* < 0.05) of the two groups was determined using the Student *t*-test, and comparison of multi-groups was determined using one-way ANOVA followed by Tukey's post-hoc test.

**Table S1 Plasma levels of the metabolites of PUFAs for BC patients and healthy controls**

| Metabolites | Control (n = 33, nM) | BC (n = 33, nM) | *P* value |
| --- | --- | --- | --- |
| PGD_2_ | 0.23±0.15 | 1.64±0.76 | *P*<0.0001 |
| PGE_2_ | 0.12±0.07 | 0.47±0.3 | *P*<0.0001 |
| TXB_2_ | 0.35±0.21 | 2.28±0.71 | *P*<0.0001 |
| 2,3-dinor-TXB_2_ | 18.77±22.19 | 4.41±1.36 | 0.0004 |
| 6-keto-PGF_1a_ | 1.09±0.7 | 3.4±1.24 | *P*<0.0001 |
| PGF_2a_ | 9.64±8.55 | 2.68±1.49 | *P*<0.0001 |
| 15-deoxy-delta12,14-PGJ_2_ | 0.16±0.11 | 0.21±0.08 | 0.0401 |
| TXB_3_ | 0.96±0.69 | 1.14±0.34 | 0.1816 |
| 19,20-DiHDPE | 1.99±0.74 | 1.7±0.71 | 0.1082 |
| 17,18-DiHETE | 3.43±1.32 | 5.23±4.1 | 0.0193 |
| 14,15-DHET | 0.68±0.21 | 0.74±0.19 | 0.2021 |
| 11,12-DHET | 0.49±0.12 | 0.5±0.18 | 0.7967 |
| 8,9-DHET | 0.35±0.15 | 0.49±0.18 | 0.0011 |
| 5,6-DHET | 0.34±0.1 | 0.46±0.22 | 0.0045 |
| 12,13-DiHOME | 5.98±2.21 | 5.87±4.34 | 0.8999 |
| 9,10-DiHOME | 5.39±2.5 | 5.21±8.24 | 0.9062 |
| 20-HETE | 1.94±3.27 | 2.73±3.18 | 0.3271 |
| 20-COOH-ARA | 3.41±2.6 | 5.33±2.97 | 0.0071 |
| 15-HETE | 1.35±0.64 | 2.08±1.05 | 0.0011 |
| 12-HETE | 6.11±5.71 | 2.08±1.05 | 0.0002 |
| 11-HETE | 0.6±0.19 | 1.07±0.7 | 0.0006 |
| 9-HETE | 0.33±0.13 | 1.11±0.87 | *P*<0.0001 |
| 8-HETE | 0.41±0.15 | 0.74±0.55 | 0.0015 |
| 13(S)-HODE | 46.66±39.93 | 51.76±40.24 | 0.6070 |
| 9(S)-HODE | 60.14±55.35 | 50.11±31.9 | 0.3707 |
| 13(S)-HOTrE | 2.26±3.41 | 1.24±1.08 | 0.1049 |
| 9(S)-HOTrE | 1.58±3.43 | 1.24±1.08 | 0.5846 |
| 13-oxo-ODE | 2.75±2.33 | 2.39±1.97 | 0.5029 |
| 9-oxo-ODE | 3.02±2.75 | 5.55±4.3 | 0.0060 |
| 15-oxo-ETE | 0.12±0.08 | 0.25±0.21 | 0.0011 |
| 5-HETE | 1.43±0.45 | 2.34±1.22 | 0.0002 |
| 19(20)-EpDPA | 0.68±0.93 | 1.36±1.31 | 0.0167 |
| 16(17)-EpDPA | 0.86±1.2 | 0.49±0.55 | 0.1099 |
| 17(18)-EpETE | 0.21±0.43 | 0.19±0.32 | 0.8452 |
| 14(15)-EET | 0.12±0.09 | 0.51±0.44 | *P*<0.0001 |
| 11(12)-EET | 0.07±0.08 | 0.35±0.3 | *P*<0.0001 |
| 8(9)-EET | 0.16±0.1 | 0.5±0.39 | *P*<0.0001 |
| 5(6)-EET | 0.72±0.45 | 1.32±1.09 | 0.0045 |
| 9(10)-EpOME | 2.98±3.98 | 11.07±9.81 | *P*<0.0001 |
| 12(13)-EpOME | 8.14±5.12 | 24.39±21.04 | 0.0001 |

Data are presented as Mean ± SD. Statistical comparison of the two groups was performed using the Student *t*-test (normal distribution) or Wilcoxon–Mann–Whitney test (nonnormal distribution). Abbreviations: 15d-PGJ_2_,15-deoxy-Δ12,14-PGJ_2_; 6-keto-PGF1α, 6-keto prostaglandin F1α; ARA, arachidonic acid; DiHDPE, dihydroxydocosapentaenoic acid; DiHETEs, dihydroxyeicosatetraenoic acids; DiHOMEs, dihydroxyoctadecenoic acids; EpETEs, epoxyeicosatetraenoic acids; EETs, epoxyeicosatrienoic acids; EpOMEs, epoxyoctadecenoicacids; EpDPA, epoxy docosapentaenoic acid; HETE, hydroxyeicosatetraenoic acid; HODE, hydroxyoctadecadienoic acid; HOTrE, hydroxyoctadeca 10,12,15-trienoic acid; hydroperoxyoctadeca-9,12-dienoicacid; oxo-ODE, oxo-octadecadienoic acid; PGD_2_, prostaglandin D_2_; PGE_1_,prostaglandin E_1_; PGE_2_, prostaglandin E_2_; PGF_2α_, prostaglandin F_2α_; PGJ_2_, prostaglandin J_2_; TXB_2_, thromboxane B_2_.

**Table S2.** **Plasma levels of the metabolites of PUFAs for the TNBC mice and controls**

| Metabolites | WT (n = 6, nM) | MMTV-PyMT (n = 6, nM) | *P* value |
| --- | --- | --- | --- |
| 13-HOTrE | 3.02±0.58 | 6.84±3.09 | 0.0139 |
| 13-oxo-ODE | 3.82±1.58 | 6.04±1.72 | 0.0420 |
| 9-oxo-ODE | 5.66±1.81 | 8.11±1.74 | 0.0372 |
| 9(s)-HOTrE | 0.95±0.16 | 2.04±1.06 | 0.0320 |
| 9(10)-EpOME | 4.2±0.78 | 7.41±0.87 | 0.0001 |
| 13-HODE | 21.24±4.03 | 38.97±15.99 | 0.0250 |
| 9-HODE | 13.84±2.68 | 26.23±12.35 | 0.0372 |
| 12(13)-EpOME | 4.86±0.78 | 7.93±1 | 0.0001 |
| 9,10-DiHOME | 9.61±5.41 | 28.87±25.64 | 0.1021 |
| 8-HEPE | 0.05±0.02 | 0.07±0.02 | 0.0744 |
| 12-HEPE | 0.45±0.25 | 0.77±0.31 | 0.0761 |
| 12-oxo-ETE | 0.22±0.2 | 0.43±0.3 | 0.1835 |
| 15-oxo-ETE | 0.33±0.16 | 0.49±0.22 | 0.1902 |
| 5-oxo-ETE | 0.31±0.13 | 0.43±0.2 | 0.2694 |
| 11-HEPE | 0.02±0.01 | 0.03±0.02 | 0.1223 |
| 19(R)-HETE | 0.31±0.11 | 0.51±0.07 | 0.0032 |
| 12-HETE | 22.63±10.79 | 49.2±19.58 | 0.0155 |
| 5-HETE | 2.09±0.37 | 2.52±0.38 | 0.0767 |
| 15-HETE | 2.09±0.37 | 2.52±0.38 | 0.0767 |
| 11-HETE | 0.75±0.2 | 1.41±0.6 | 0.0279 |
| 8-HETE | 1.06±0.32 | 1.76±0.43 | 0.0094 |
| 9-HETE | 0.51±0.17 | 0.87±0.34 | 0.0437 |
| 11(12)-EET | 0.2±0.04 | 0.34±0.17 | 0.0790 |
| 14(15)-EET | 0.71±0.09 | 1.12±0.55 | 0.1011 |
| 8(9)-EET | 0.66±0.15 | 1.11±0.44 | 0.0385 |
| 5(6)-EET | 10.49±2.7 | 11.05±2.35 | 0.7111 |
| 9,10,13-TriHOME | 0.39±0.3 | 0.88±0.76 | 0.1684 |
| 9,12,13-TriHOME | 3.96±0.72 | 5.55±2.67 | 0.1883 |
| 20-Carboxy-AA | 8.19±2.6 | 8.24±1.24 | 0.9656 |
| 17,18-DiHETE | 0.49±0.14 | 0.69±0.3 | 0.1545 |
| 11,12-DiHET | 0.24±0.06 | 0.51±0.2 | 0.0106 |
| 14,15-DiHET | 0.46±0.13 | 0.84±0.17 | 0.0014 |
| 8,9-DiHET | 1.37±0.37 | 1.62±0.26 | 0.2060 |
| 5,6-DiHET | 1.05±0.2 | 1.06±0.2 | 0.9804 |
| 13,14-EpDPA | 0.25±0.09 | 0.36±0.07 | 0.0523 |
| 4-HDHA | 1.02±0.24 | 1.21±0.48 | 0.3913 |
| 8-HDHA | 0.85±0.2 | 1.09±0.24 | 0.0846 |
| 10-HDHA | 0.29±0.12 | 0.49±0.14 | 0.0244 |
| 16,17-EpDPA | 0.32±0.07 | 0.35±0.13 | 0.6083 |
| 10(11)-EpDPA | 0.32±0.09 | 0.34±0.1 | 0.6765 |
| 13-HDHA | 0.11±0.07 | 0.26±0.1 | 0.0158 |
| 16-HDHA | 0.22±0.07 | 0.36±0.13 | 0.0355 |
| EKODE | 5.61±3.07 | 8.53±4.08 | 0.1922 |
| 12,13-DiHOME | 7.37±2.42 | 18.87±12.21 | 0.0472 |
| 20-HEPE | 0.41±0.12 | 0.75±0.24 | 0.0103 |
| 18-HEPE | 0.08±0.05 | 0.18±0.04 | 0.0040 |
| 17(18)-EpETE | 0.09±0.03 | 0.13±0.04 | 0.1337 |
| 9-HEPE | 0.04±0.02 | 0.05±0.02 | 0.4355 |
| 20-HETE | 0.62±0.17 | 0.94±0.25 | 0.0286 |
| 19(R)-HETE | 0.25±0.12 | 0.44±0.11 | 0.0176 |
| 5,15-DiHETE | 0.06±0.04 | 0.06±0.08 | 0.9680 |
| 19(20)-EpDPA | 1.94±0.57 | 2.68±0.72 | 0.0749 |
| 7(8)-EpDPA | 0.34±0.11 | 0.39±0.13 | 0.4828 |
| 22-HDHA | 2.79±0.63 | 4.77±0.41 | 0.0001 |
| 14-HDHA | 2.33±1.03 | 4.37±2.04 | 0.0530 |
| 7-HDHA | 0.32±0.1 | 0.4±0.12 | 0.2754 |
| 20-HDHA | 0.58±0.19 | 0.79±0.14 | 0.0497 |
| 17-HDHA | 0.23±0.08 | 0.41±0.08 | 0.0044 |
| 11-HDHA | 0.21±0.06 | 0.27±0.05 | 0.0580 |
| PGE_3_ | 4.04±1.92 | 4.58±2.15 | 0.6552 |
| 13,14-didydro-15-keto PGE_2_ | 0.6±0.14 | 1.42±1.29 | 0.1529 |
| PGE_2_ | 0.19±0.08 | 0.37±0.11 | 0.0108 |
| PGD_2_ | 0.25±0.06 | 0.38±0.18 | 0.1279 |
| 7,8-DiHDPA | 0.72±0.2 | 0.74±0.14 | 0.9046 |
| 16,17-DiHDPA | 0.35±0.09 | 0.55±0.12 | 0.0062 |
| 13,14-DiHDPA | 0.21±0.05 | 0.36±0.1 | 0.0070 |
| 19,20-DiHDPA | 2.66±0.76 | 3.79±0.78 | 0.0287 |
| 10,11-DiHDPA | 0.16±0.05 | 0.24±0.08 | 0.0717 |
| TXB_2_ | 0.74±0.46 | 1.99±1.98 | 0.1625 |

Data are presented as Mean ± SD. Statistical comparison of the two groups was performed using Student t-test (normal distribution) or Wilcoxon–Mann–Whitney test (nonnormal distribution).

Abbreviations: 5,15-DiHETE, 5,15-Dihydroxyeicosatetraenoic acid; 9,10,13-TriHOME, 9,10,13-Trihydroxy-11-octadecenoic acid; 9,12,13-TriHOME, 9(S), 12(S), 13(S)-Trihydroxy-10(e)-octadecenoic acid; DiHETrEs, dihydroxyeicosatrienoic acids; EpDPEs, epoxydocosapentaenoic acids; EpFAs, epoxy fatty acids; EpODEs, epoxyoctadecadienoic acids; HEDH, hydroxyeicosanoid dehydrogenase; HDHA, hydroxy docosahexaenoic acid; HEPE, hydroxyeicosapentaenoic acid.

**Table S3. Plasma levels of the metabolites of PUFAs for the TNBC mice and the TNBC mice receiving Clo and SKF treatment (to be continued)**

| Metabolites | Controls  (n = 9, nM) | TNBC  (n= 9, nM) | TNBC + Clo  (n= 8, nM) | TNBC + SKF  (n= 9, nM) |
| --- | --- | --- | --- | --- |
| 13-HOTrE | 3.5±1.02 | 6.69±3.21* | 5.36±2.95 | 4.77±2.6 |
| 13-oxo-ODE | 3.98±1.59 | 6.74±2.65* | 5.93±2.28 | 4.85±2.04 |
| 9-oxo-ODE | 5.72±1.95 | 8.54±3.01* | 7.64±2.43 | 6.86±2.41 |
| 9(s)-HOTrE | 1.01±0.18 | 1.72±0.92* | 1.31±0.7 | 1.17±0.4 |
| 9(10)-EpOME | 3.84±1.03 | 6.7±1.98** | 4.73±1.07^^ | 4.18±1.44^^ |
| 13-HODE | 22.53±4.24 | 34.55±15* | 27.7±14.06 | 24.37±9.6 |
| 9-HODE | 14.49±2.99 | 23.86±10.68** | 22.01±11.89 | 17.49±6.75 |
| 12(13)-EpOME | 4.49±1.13 | 7.29±1.82* | 5.6±1.26^ | 4.61±1.55^^ |
| 9,10-DiHOME | 9.55±4.31 | 19.59±22.62 | 4.33±2.67** | 6.03±3.7 |
| 8-HEPE | 0.06±0.03 | 0.07±0.02 | 0.05±0.04 | 0.07±0.03 |
| 12-HEPE | 0.51±0.26 | 0.91±0.46* | 1.25±0.74* | 0.91±0.77 |
| 12-oxo-ETE | 0.21±0.18 | 0.42±0.26 | 0.75±0.48** | 0.57±0.61 |
| 15-oxo-ETE | 0.37±0.17 | 0.56±0.2* | 0.43±0.27 | 0.31±0.18^ |
| 5-oxo-ETE | 0.33±0.13 | 0.47±0.17 | 0.36±0.2 | 0.26±0.13^^ |
| 11-HEPE | 0.02±0.02 | 0.04±0.02 | 0.05±0.04 | 0.03±0.03 |
| 19(R)-HETE | 0.38±0.16 | 0.59±0.31 | 0.46±0.08 | 0.35±0.14 |
| 12-HETE | 22.43±9.01 | 45.03±18.06** | 72.35±56.26* | 56.27±59.19 |
| 5-HETE | 2.21±0.46 | 2.47±0.35 | 1.64±0.55*^^ | 2.03±0.51^ |
| 15-HETE | 2.21±0.46 | 2.47±0.35 | 1.64±0.55*^^ | 2.03±0.51^ |
| 11-HETE | 0.85±0.24 | 1.32±0.48* | 1.65±1.2 | 1.23±0.61 |
| 8-HETE | 1.11±0.3 | 1.73±0.4** | 1.4±0.68 | 1.63±0.66* |
| 9-HETE | 0.54±0.17 | 0.81±0.27* | 0.59±0.4 | 0.57±0.31 |
| 11(12)-EET | 0.19±0.04 | 0.31±0.14* | 0.19±0.06^ | 0.21±0.09 |
| 14(15)-EET | 0.68±0.12 | 1.04±0.43* | 0.66±0.22^ | 0.79±0.29 |
| 8(9)-EET | 0.62±0.16 | 1.04±0.36** | 0.63±0.21^ | 0.74±0.32 |
| 5(6)-EET | 9.69±2.72 | 9.52±2.64 | 8.9±1.08 | 8.11±2.02 |
| 9,10,13-TriHOME | 0.52±0.41 | 1.03±1.02 | 1.43±1.53 | 0.78±0.69 |
| 9,12,13-TriHOME | 4.64±1.54 | 5.99±3.23 | 7.27±3.86 | 4.9±2.06 |
| 20-Carboxy-ARA | 8.18±2.12 | 8.25±1.98 | 6.5±1.07^ | 7.52±1.26 |
| 17,18-DiHETE | 0.61±0.25 | 0.71±0.33 | 0.67±0.48 | 0.84±0.98 |
| 11,12-DiHET | 0.3±0.11 | 0.47±0.2* | 0.35±0.3 | 0.48±0.63 |
| 14,15-DiHET | 0.59±0.24 | 0.84±0.31 | 0.87±0.79 | 1.1±1.83 |
| 8,9-DiHET | 1.37±0.38 | 1.61±0.25 | 0.85±0.39*^^^ | 1.2±0.52^ |
| 5,6-DiHET | 1.04±0.2 | 1.05±0.19 | 0.54±0.16***^^^ | 0.75±0.26*^ |
| 13,14-EpDPA | 0.23±0.09 | 0.36±0.07** | 0.2±0.08^^^ | 0.22±0.04^^^^ |
| 4-HDHA | 1.09±0.3 | 1.31±0.38 | 0.94±0.39 | 0.9±0.26^ |
| 8-HDHA | 0.9±0.24 | 1.14±0.22* | 0.72±0.36^^ | 0.77±0.2^^ |
| 10-HDHA | 0.32±0.13 | 0.53±0.22* | 0.58±0.57 | 0.47±0.3 |

**Table S3. Plasma levels of the metabolites of PUFAs for the TNBC mice and the TNBC mice receiving Clo and SKF treatment (continued)**

| Metabolites | Controls  (n = 9, nM) | TNBC  (n= 9, nM) | TNBC + Clo  (n= 8, nM) | TNBC + SKF  (n= 9, nM) |
| --- | --- | --- | --- | --- |
| 16,17-EpDPA | 0.31±0.08 | 0.38±0.11 | 0.27±0.07^ | 0.26±0.08^ |
| 10(11)-EpDPA | 0.3±0.09 | 0.34±0.08 | 0.26±0.08^ | 0.25±0.07^ |
| 13-HDHA | 0.13±0.09 | 0.26±0.09 ** | 0.37±0.43 | 0.21±0.24 |
| 16-HDHA | 0.25±0.1 | 0.37±0.11 * | 0.45±0.44 | 0.26±0.15 |
| EKODE | 5.86±2.98 | 9.66±4.7 | 6.91±2.79 | 5.38±2.34^ |
| 12,13-DiHOME | 8.15±2.34 | 15.26±11.18 | 6.82±2.38 | 6.21±3.03^ |
| 20-HEPE | 0.57±0.29 | 0.7±0.26 | 0.57±0.28 | 0.56±0.47 |
| 18-HEPE | 0.1±0.06 | 0.18±0.07 * | 0.12±0.08 | 0.1±0.08^ |
| 17(18)-EpETE | 0.09±0.04 | 0.15±0.06 * | 0.11±0.05 | 0.09±0.04^ |
| 9-HEPE | 0.04±0.02 | 0.05±0.02 | 0.05±0.02 | 0.06±0.02 |
| 20-HETE | 0.73±0.23 | 0.9±0.23 | 0.81±0.3 | 0.42±0.3*^^ |
| 19(R)-HETE | 0.32±0.16 | 0.51±0.34 | 0.35±0.09 | 0.3±0.15 |
| 5,15-DiHETE | 0.08±0.05 | 0.06±0.06 | 0.08±0.12 | 0.05±0.04 |
| 19(20)-EpDPA | 2.11±0.66 | 2.97±1.22 | 1.95±0.48^ | 1.72±0.61^ |
| 7(8)-EpDPA | 0.34±0.12 | 0.38±0.1 | 0.28±0.09^ | 0.28±0.1^ |
| 22-HDHA | 3.43±1.21 | 4.88±1.3 * | 4.09±2.05 | 3.27±1.88^ |
| 14-HDHA | 2.47±0.93 | 5.1±2.6 * | 5.04±3.17* | 4.46±3.67 |
| 7-HDHA | 0.35±0.1 | 0.41±0.09 | 0.27±0.18^ | 0.34±0.12 |
| 20-HDHA | 0.68±0.29 | 0.89±0.2 | 0.82±0.55 | 0.65±0.44 |
| 17-HDHA | 0.28±0.11 | 0.42±0.08 ** | 0.54±0.47 | 0.4±0.33 |
| 11-HDHA | 0.22±0.07 | 0.29±0.05 * | 0.28±0.21 | 0.22±0.11 |
| PGE3 | 5.5±3.99 | 12.44±25.48 | 4.94±6.96 | 3.66±5.25 |
| 13,14-didydro-15-keto PGE_2_ | 0.69±0.26 | 1.25±1.02 | 1.38±1.31 | 0.72±0.24 |
| PGE_2_ | 0.26±0.13 | 0.36±0.12 | 0.7±0.88 | 0.44±0.47 |
| PGD_2_ | 0.29±0.11 | 0.38±0.16 | 0.59±0.54 | 0.48±0.57 |
| 7,8-DiHDPA | 0.7±0.17 | 0.73±0.11 | 0.37±0.2**^^^^ | 0.54±0.13*^^ |
| 16,17-DiHDPA | 0.45±0.18 | 0.55±0.21 | 0.53±0.43 | 0.71±1.01 |
| 13,14-DiHDPA | 0.26±0.11 | 0.37±0.18 | 0.3±0.27 | 0.45±0.67 |
| 19,20-DiHDPA | 3.17±1.1 | 4.29±2.05 | 4.13±2.07 | 3.79±3.62 |
| 10,11-DiHDPA | 0.19±0.06 | 0.24±0.08 | 0.15±0.13 | 0.21±0.17 |
| TXB_2_ | 0.78±0.46 | 1.67±1.55 | 2.25±1.27** | 1.71±1.37 |

Data are presented as Mean ± SD. Statistical difference was determined by one-way ANOVA followed by Tukey's or Games-Howell post-hoc comparison test. Note, ****P* < 0.001, ***P* < 0.01, **P* < 0.05 VS. control group; ^^^ *P* < 0.001, ^^ *P* < 0.01, **^** *P* < 0.05 VS. TNBC group.

**Table S4** shRNA Sequences in this study

|  | shRNA | Sequences |
| --- | --- | --- |
| shCYP2J2#1 | CYP2J2S1 | GATCGAACTTCTTCCTTGTGGACTTCCTCGAGGAAGTCCACAAGGAAGAAGTTTTTTTG |
|  | CYP2J2S2 | AATTCAAAAAAACTTCTTCCTTGTGGACTTCCTCGAGGAAGTCCACAAGGAAGAAGTTC |
| shCYP2J2#2 | CYP2J2S3 | GATCGAAGTCACATACTTGGAGGCTTCTCGAGAAGCCTCCAAGTA TGTGACTTTTTTTG |
|  | CYP2J2S4 | AATTCAAAAAAAGTCACATACTTGGAGGCTTCTCGAGAAGCCTCCAAGTATGTGACTTC |
| shCYP2J2#3 | CYP2J2S5 | GATCGAAGTTTAGAATGGGTATCACCCTCGAGGGTGATACCCATT CTAAACTTTTTTTG |
|  | CYP2J2S6 | AATTCAAAAAAAGTTTAGAATGGGTATCACCCTCGAGGGTGATACCCATTCTAAACTTC |
| shCXCL9#1 | CXCL9S1 | GATCGAAAGGGTCGCTGTTCCTGCATCTCGAGATGCAGGAACAGCGACCCTTTTTTTTG |
|  | CXCL9S2 | AATTCAAAAAAAAGGGTCGCTGTTCCTGCATCTCGAGATGCAGGAACAGCGACCCTTTC |
| shCXCL9#2 | CXCL9S3 | GATCGAACCCAGATTCAGCAGATGTGCTCGAGCACATCTGCTGAATCTGGGTTTTTTTG |
|  | CXCL9S4 | AATTCAAAAAAACCCAGATTCAGCAGATGTGCTCGAGCACATCTGCTGAATCTGGGTTC |
| shCXCL9#3 | CXCL9S5 | GATCGAAGAAGACTACATAAGAGACCCTCGAGGGTCTCTTATGTAGTCTTCTTTTTTTG |
|  | CXCL9S6 | AATTCAAAAAAAGAAGACTACATAAGAGACCCTCGAGGGTCTCTTATGTAGTCTTCTTC |
| shPLEC#1 | PLECS1 | GATCGAATGGAAGCAGTGGCTCGCCCCTCGAGGGGCGAGCCACTGCTTCCATTTTTTTG |
|  | PLECS2 | AATTCAAAAAAATGGAAGCAGTGGCTCGCCCCTCGAGGGGCGAGCCACTGCTTCCATTC |
| shPLEC#2 | PLECS3 | GATCGAAGTGGGTCAACAAGCACCTCCTCGAGGAGGTGCTTGTTGACCCACTTTTTTTG |
|  | PLECS4 | AATTCAAAAAAAGTGGGTCAACAAGCACCTCCTCGAGGAGGTGCTTGTTGACCCACTTC |
| shPLEC#3 | PLECS5 | GATCGAACTGCTGCAGCAGCAGAAGGCTCGAGCCTTCTGCTGCTGCAGCAGTTTTTTTG |
|  | PLECS6 | AATTCAAAAAAACTGCTGCAGCAGCAGAAGGCTCGAGCCTTCTGCTGCTGCAGCAGTTC |

shRNA sequences were synthesized and inserted into the pLVX-GFP+PURO-3xFlag vector. Highlighted shRNAs had the most significant knockdown effect and were used in follow-up studies, as detailed in Fig. S3, S4 and S6.

**Table S5** Chemicals and materials in this study

| Chemicals and materials | Company |
| --- | --- |
| GAPDH Polyclonal antibody (60004-1-Ig) | Proteintech, China |
| CYP2J2 Polyclonal antibody (13562-1-AP) | Proteintech, China |
| PLEC Polyclonal antibody (29170-1-AP) | Proteintech, China |
| NFKB1 Monoclonal antibody (66992-1-Ig) | Proteintech, China |
| CXCL9 Polyclonal antibody(ab263442) | Abcam, USA |
| Anti-CYP2C8 (ab88904) | Abcam, USA |
| Anti-CYP2C19 (ab137015) | Abcam, USA |
| Anti-CYP2C9 (ab150364) | Abcam, USA |
| CYP2C18 antibody (GTX33135) | GeneTex, USA |
| Goat Anti-Rabbit IgG H&L (550090) | Zenbioscience, China |
| 12(13)-EpOME (No. 52450) | Cayman Chemical, USA |
| Clotrimazole (HY-10882) | MedChemExpress, China |
| proadifen hydrochloride (HY-B1311) | MedChemExpress, China |
| MDA-MB-231 (CRM-HTB-26) | ATCC, USA |
| MDA-MB-436 (HTB-130) | ATCC, USA |
| MDA-MB-157 (HTB-24) | ATCC, USA |
| BT-549 (HTB-122) | ATCC, USA |
| MCF-7 (CRL-3435) | ATCC, USA |
| BT-474 (HTB-20) | ATCC, USA |
| MCF-10A (CRL-10317) | ATCC, USA |
| HEK293T (CRL-1573) | ATCC, USA |

**Table S6** Primer sequences used in this study

| Gene | Forward primer | Reverse primer |
| --- | --- | --- |
| *Gapdh* | AATGGATTTGGACGCATTGGT | TTTGCACTGGTACGTGTTGAT |
| *Cyp2j5* | CAACACTGCGATGGGCTTTG | GACTCTCGGTCAGACAAGCTC |
| *Cyp2j6* | TTAGCCACGATCTGGGCAG | CTGGGGGATAGTTCTTGGGG |
| *Cyp2j8* | ACCCAACCATAGATGACCGAG | GCGACGCCCATTTTGAAATTC |
| *Cyp2j9* | ATGCGCCTTCCTTTCGTGG | CCAGGCTTAGAACATTCCCGTA |
| *Cyp2c39* | GAGGAAGCATTCCAATGGTAGAA | TGTGAAGCGCCTAATCTCTTTC |
| *Cyp2c40* | GAGGAAGCATTCCAATGGTA | TGTGAAGCGCCTAATCTCTTT |
| *Fasn* | GGCTCTATGGATTACCCAAGC | CCAGTGTTCGTTCCTCGGA |
| *PPARγ* | GGAAGACCACTCGCATTCCTT | GTAATCAGCAACCATTGGGTCA |
| *Fabp4* | ATCAGCGTAAATGGGGATTTGG | GTCTGCGGTGATTTCATCGAA |
| *Cebpa* | CAAGAACAGCAACGAGTACCG | GTCACTGGTCAACTCCAGCAC |
| *Srebf1* | TGACCCGGCTATTCCGTGA | CTGGGCTGAGCAATACAGTTC |
| *CYP2C8* | CATTACTGACTTCCGTGCTACAT | CTCCTGCACAAATTCGTTTTCC |
| *CYP2C18* | GGAAAACGGATGTGTATGGGAG | GTGGCACACGACCAAATGC |
| *CYP2C19* | GGAAAACGGATTTGTGTGGGA | GGTCCTTTGGGTCAATCAGAGA |
| *CYP2J2* | TGGCTTGCCCTTAATCAAAGAA | GGCCACTTGACATAATCAATCCA |
| *GAPDH* | TGTGGGCATCAATGGATTTGG | ACACCATGTATTCCGGGTCAAT |
| *CXCL9* | CCAGTAGTGAGAAAGGGTCGC | AGGGCTTGGGGCAAATTGTT |
| *PLEC* | TCCTCTTTGACAAAACACTGCT | TGAGTCCAGTTCCGTTTCTCC |
| ChIP primer | GGACATGCCCTTTTTCTGCC | ACCATCTCTAGATTGCTGCCC |

1. Correspondence should be addressed to Prof. Jun-Yan Liu, [Tel: +86-23-6848](mailto:Tel:%20+86-23-6848) 3587; email: jyliu@cqmu.edu.cn; ORCID ID: 0000-0002-3018-0335 [↑](#footnote-ref-1)
