# Supplementary figures and images for: Epoxy metabolites of linoleic acid promote the development of breast cancer via orchestrating PLEC/NFκB1/CXCL9-mediated tumor growth and metastasis
Source: Cell Death Dis. 2024 Dec 18;15(12):901. doi: 10.1038/s41419-024-07300-6 (PMC11655665; doi:10.1038/s41419-024-07300-6)

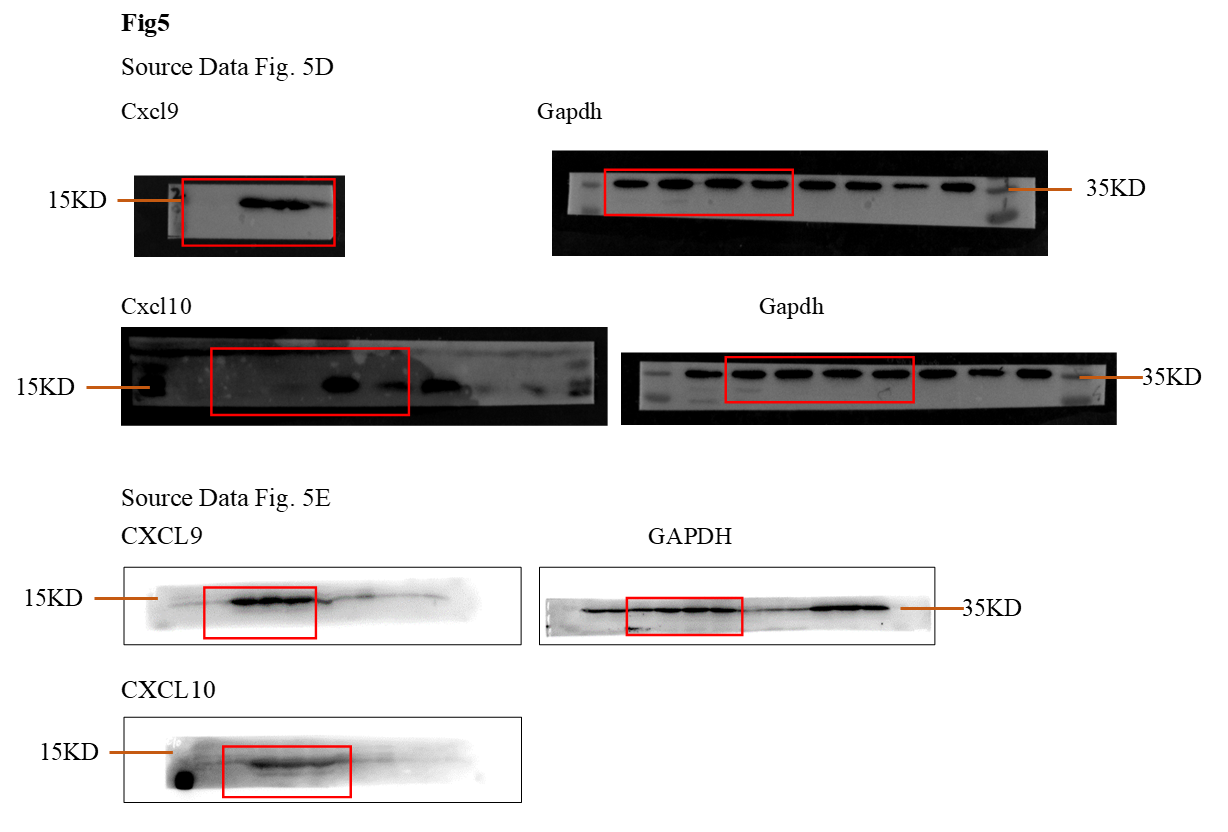


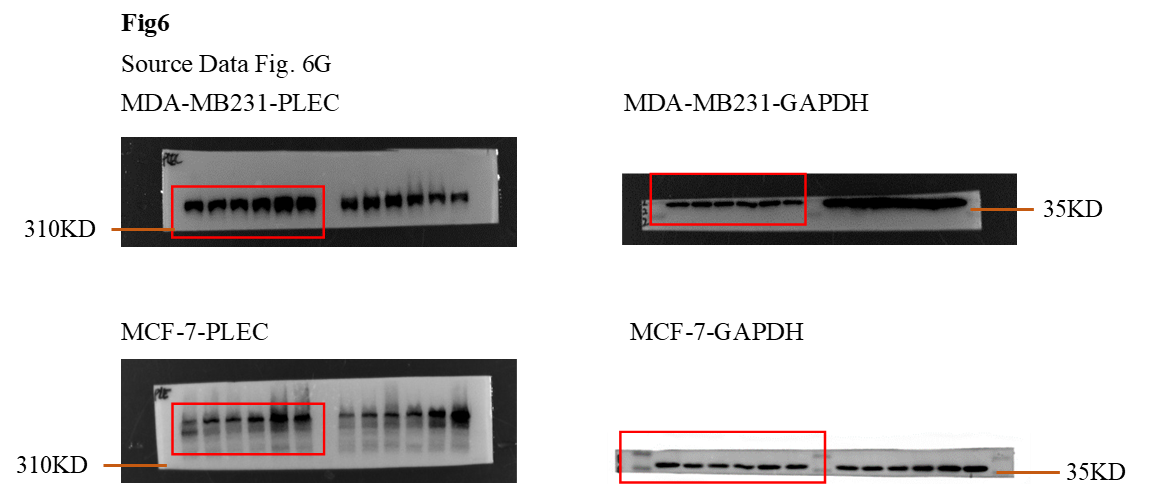


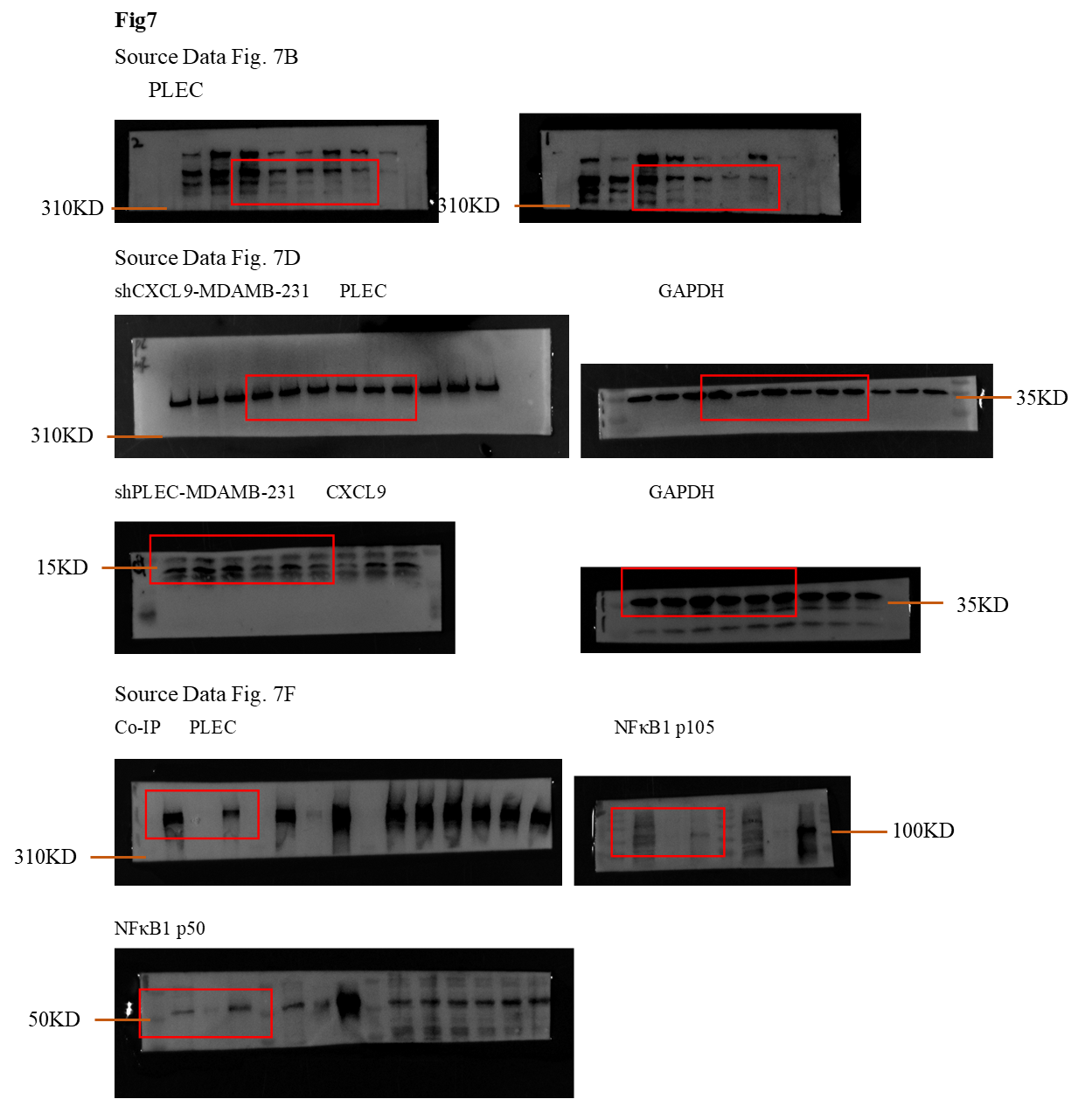


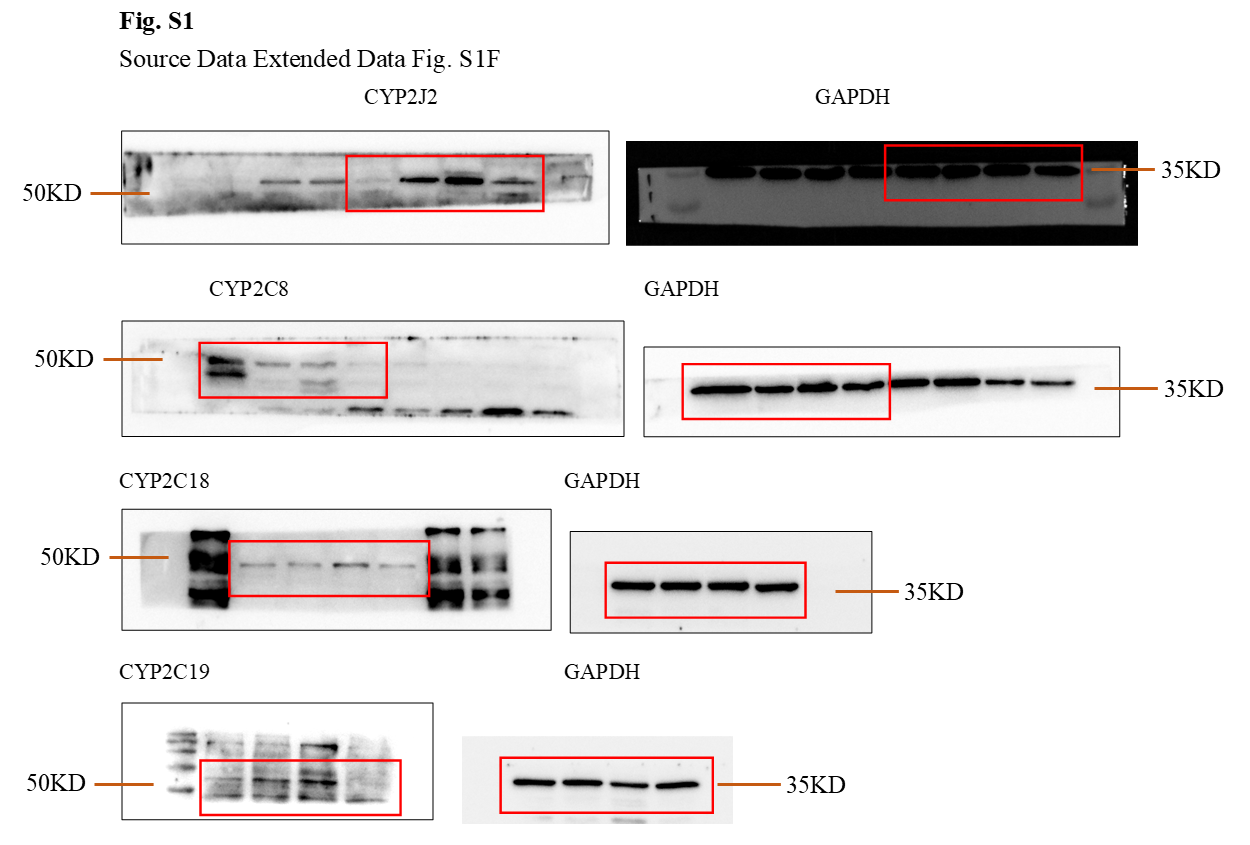


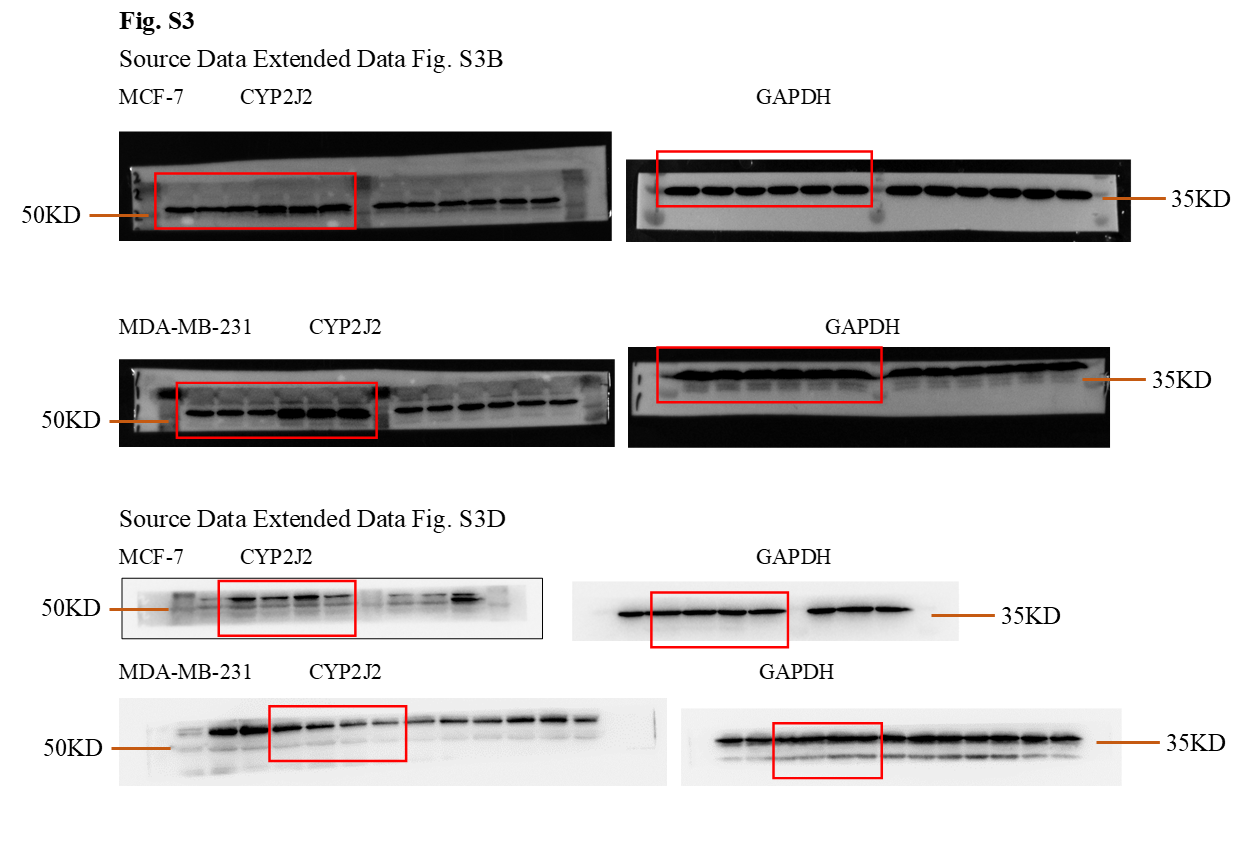


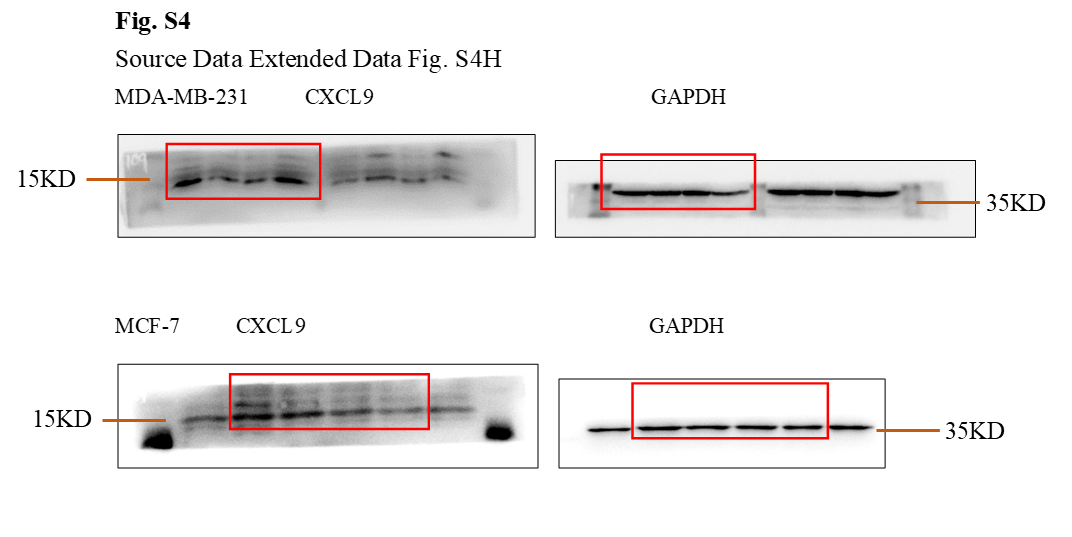


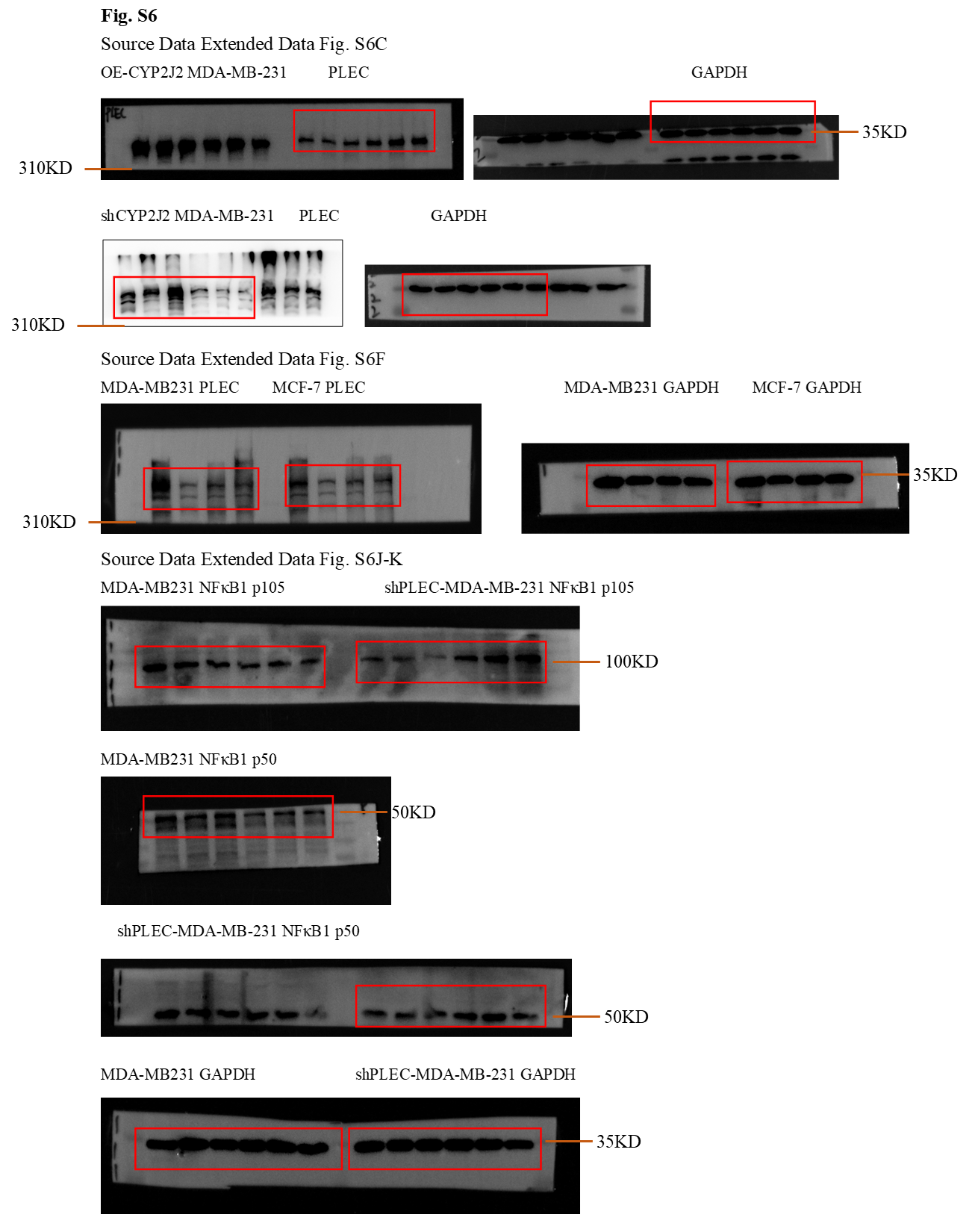

Supplement: Supplementary file 2 — Original western blots. [file 41419_2024_7300_MOESM2_ESM.docx]
